# Incorporating Gold Nanoparticles with Varying Diameters into Freely Floating Nanosheets via a Biphasic Monolayer Adsorption Assembly Mechanism

**DOI:** 10.1021/acs.langmuir.5c04350

**Published:** 2025-12-10

**Authors:** Ellen J. Robertson, Chao Yang, Antonia Sofia Soto Carrillo, C’Lannye James, Christopher B. Whitehead

**Affiliations:** a Chemistry Department, 7254 Union College, 807 Union St., Schenectady, New York 12308, United States; b Materials Science and Engineering Department, 8024 Rensselaer Polytechnic Institute, Troy, New York 12180, United States

## Abstract

Peptoid nanosheets are versatile two-dimensional nanomaterials that can form through the assembly and collapse of peptoid monolayers at fluid interfaces. The resulting material freely floats in water due to its hydrophilic exterior (which can be functionalized to bind targets of interest) and hydrophobic interior. The oil–water interface is a rich environment for nanosheet synthesis, as it allows for hydrophobic cargo dispersed in the oil phase to be incorporated into the nanosheet interior. In this work, we describe the synthesis and characterization of organic–inorganic hybrid nanosheets formed using octadecanethiol-functionalized gold nanoparticles with a wide range of particle diameters (∼10–80 nm) via this unique biphasic monolayer adsorption assembly and collapse mechanism at the toluene–water interface. We demonstrate through optical microscopy, atomic force microscopy, and scanning electron microscopy measurements that a range of particle sizes can be successfully embedded between two peptoid monolayers. The particles are arranged in a single patchy monolayer within the nanosheets. The findings presented here open the door for creating multifunctional hybrid peptoid nanosheets that not only bind targets of interest but also possess useful optical, electronic, catalytic, and/or magnetic properties.

## Graphical abstract

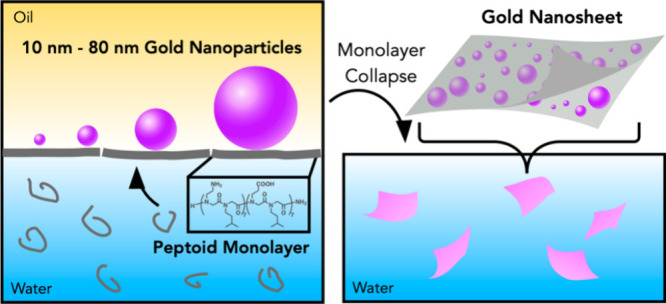

## Introduction

Hybrid nanostructures formed with organic and inorganic components are fascinating materials with potential widespread uses, such as in drug delivery,^1^ catalysis,^2^ and sensing^3^ applications. Hybrid nanostructures formed from organic polymers and inorganic nanoparticles offer beneficial properties from each component, such as biocompatibility and targeted binding ability from the polymer and interesting electronic, optical, and/or magnetic properties from the inorganic nanoparticles.^1^ Hybrid two-dimensional (2D) nanostructures are of particular interest as the confinement of nanoparticles into a 2D architecture can lead to enhanced properties. For instance, metal nanoparticles in proximity experience coupling of localized surface plasmon resonances (LSPRs) on adjacent nanoparticles.^4^ This LSPR coupling phenomenon results in the generation of an intense light-induced electromagnetic field that is localized to the region between adjacent nanoparticles, which is beneficial in sensing^4^ and catalysis^5^ applications. Moreover, the 2D polymer platform has the potential to act as a vehicle for the delivery of therapeutic particles.^1^

Hybrid polymer-inorganic 2D nanostructures are typically formed using layer-by-layer assembly techniques, which can produce highly ordered and ultrathin 2D materials.^6,7^ Layer-by-layer assembly techniques are limited, however, because they often require that the material is templated on a solid substrate.^6^ The presence of the solid substrate limits the flexibility of the hybrid nanomaterials and their ability to be used in solution-based applications.^8^ Removing the materials from the substrates poses challenges such as damaging the material, while colloidal-based synthesis methods can often lead to materials that lack well-defined order.^7^

One strategy to overcome these limitations associated with assembling hybrid 2D nanomaterials is to use freely floating 2D arrays of sequence defined polymers^9^ as platforms for nanoparticle assembly. For example, peptide sequences have been designed to both assemble into 2D nanostructures and immobilize gold nanostructures on their surfaces.^10^ Self-assembled amphiphilic DNA nanosheets have also served as platforms for assembling gold nanoparticles.^11^ In addition, 2D synthetic polymer assemblies have been used to template nanoparticles, in which metal nanoparticles are incorporated into block copolymer two-dimensional crystalline assemblies functionalized with oxygen-containing functional groups.^12^ Using biological and polydisperse synthetic polymers in hybrid nanoassemblies has both benefits and drawbacks. For example, peptide and DNA polymers can be precisely designed and functionalized but can be unstable outside physiological conditions and in the presence of enzymes. Conversely, synthetic block copolymers are robust but lack the sequence specificity and functionality found in biological polymers. Peptoid polymers^13^ offer a unique solution to the limitations of biological polymers and polydisperse block copolymers in the formation of 2D freely floating hybrid polymer–nanoparticle assemblies.

Peptoids^13^ are a powerful class of peptidomimetics that can be engineered to self-assemble into nanomaterials.^14–16^ Like peptides, peptoids are sequence-defined polymers in which the monomers are connected via amide bonds. With peptoids, the functional groups are attached to the backbone nitrogens rather than the backbone alpha carbons. Thus, peptoid assembly is dictated by the identities and patterning of the functional groups on the backbone rather than through backbone N–H hydrogen bonding.^17^

Several peptoid sequences have demonstrated the ability to assemble into two-dimensional (2D) nanostructures that freely float in solutions.^15,16,18,19^ These sequences take advantage of precisely positioning hydrophobic and polar residues into different block patterns. Previous work has shown that amphiphilic sequences in which one peptoid block is composed of polar ethyleneoxy side chains and the other block is composed of either hydrophobic *n*-decyl or phenyl side chains that will assemble into nanosheets in solution upon solvent evaporation.^14,18–20^ The Chen group has also designed a variety of amphiphilic block peptoid sequences that assemble into nanosheets in bulk solution, which have been studied for self-repairing membrane properties,^21^ mineralization and carbon dioxide sequestration,^22,23^ catalysis and sensing,^24^ and self-assembly through silica nanoparticle binding.^25^ Another class of peptoid nanosheets forms via the assembly and collapse of amphiphilic peptoid monolayers at fluid interfaces.^15,26,27^ These peptoid sequences display alternating polar and hydrophobic residues, with one block consisting of positively charged 2-ethylamine side chains (Nae) and the other block consisting of negatively charged 2-carboxylethyl side chains (Nce). Several hydrophobic side chains can be used in these nanosheet-forming peptoid sequences, including aromatic *N*-(2-phenylethyl)­glycine residues (Npe) and aliphatic *N*-isoamylglycine residues (Nmbu).^28,29^

The peptoid monolayer collapse mechanism is advantageous as it allows for functionalization of the peptoid nanosheet. Previous work demonstrated the functionalization of the nanosheet hydrophilic exterior. Because their surface is rich in amine and carboxylate groups, peptoid nanosheets have been used as templates for calcium carbonate mineralization.^30^ Other works have demonstrated that hydrophilic “loops” incorporated into the middle of the peptoid sequence are displayed on the surfaces of peptoid nanosheets due to monolayer compression.^31,32^ These loops have been functionalized with antibody mimetics^31^ and sugar moieties,^32^ allowing the nanosheets to bind targets of interest with high specificity and multivalency.

Not only does the monolayer collapse mechanism allow for functionalizing the nanosheet exterior, but “cargo” can also be loaded into the hydrophobic nanosheet interior when formed at the oil–water interface. A biphasic monolayer adsorption assembly mechanism can be implemented in which the cargo is dispersed in the oil phase and assembles on the transiently exposed hydrophobic face of the intermediate peptoid monolayer. The composite monolayer then collapses upon compression, trapping the cargo into the middle of the nanosheet bilayer. We previously reported on exploiting this mechanism to form freely floating 2D plasmonic nanosheets embedded with 5 nm dodecanethiol functionalized gold nanoparticles (DDT-AuNPs), which are about twice as thick as a bare peptoid nanosheet.^33,34^

In this work, we expand our biphasic monolayer adsorption assembly mechanism to a range of differently sized hydrophobic cargo (∼10–80 nm) that is much larger than the thickness of a bare nanosheet. The ability to make nanosheets with a variety of nanoparticle sizes is important for customizing the material toward specific applications. For instance, SERS sensors typically function better with plasmonic nanoparticles with diameters between 20 and 70 nm,^35,36^ particles with diameters between 2 and 6 nm can more effectively penetrate cells for drug delivery applications,^37^ and iron oxide nanoparticles with diameters between 10 and 20 nm demonstrate superparamagnetism, making them responsive to external magnetic fields.^37^ This study on incorporating larger nanoparticles into the nanosheet demonstrates how material applications can be expanded. Moreover, it tests the structural integrity of the peptoid monolayer intermediate and whether the addition of large nanoparticles to its hydrophobic face disrupts the nanosheet assembly mechanism.

To prepare the hydrophobic cargo, we functionalized citrate-AuNPs with octadecanethiol (ODT) ligands via a phase transfer process^15,18–20,18–20,38–43^ facilitated by sonication (Figure 1A). This hydrophobic functionalization rendered the AuNPs soluble in an organic solvent, toluene. We then prepared peptoid nanosheets with the ODT-AuNPs by rotating vials containing the peptoid (Nae-Nmbu)_7_-(Nce-Nmbu)_7_ ^29^ and AuNP solutions, thus inducing collapse of the composite peptoid-AuNP monolayer (Figure 1B).^33,34^ Through light microscopy, atomic force microscopy (AFM), and scanning electron microscopy (SEM) studies, we show the successful incorporation of these ODT-AuNPs into peptoid nanosheets. When the AuNPs are embedded in the peptoid nanosheet interior, the nanosheet exterior remains available for functionalization. These AuNP nanosheets are thus distinct from those in which the nanoparticles decorate the nanosheet exterior.^25^ The ability to load a wide range of differently sized cargo (a few nanometers up to hundreds of nanometers) into the peptoid nanosheet opens the door for creating new multifunctional 2D nanomaterials with interesting optical, electronic, and magnetic properties. By carefully selecting the appropriate nanoparticle size and core material as well as the peptoid hydrophilic sequence, peptoid nanosheets can be prepared with properties that are ideal for drug delivery, catalysis, and sensing applications.

**Figure 1:**
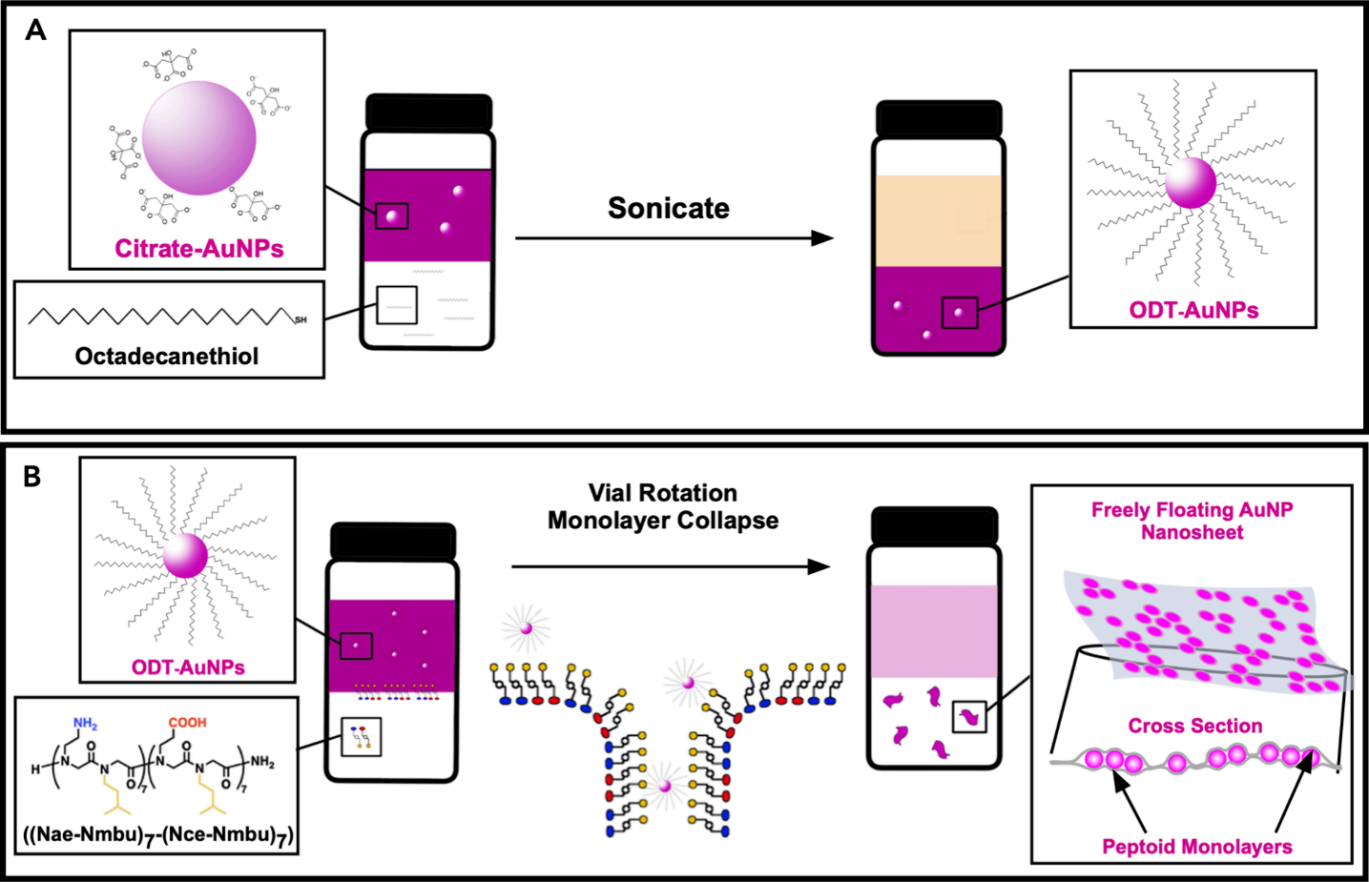
Cartoons demonstrating (A) the process used to functionalize the AuNPs with hydrophobic ODT ligands and (B) the formation of freely floating AuNP nanosheets via the biphasic monolayer adsorption assembly mechanism in which vial rotation induces the collapse of a composite peptoid-AuNP monolayer. Drawings are not to scale.

## Experimental Section

### AuNP Ligand Exchange

The ligand exchange protocol is based on previous studies.^38–43^ A ∼0.4 mM solution of ODT (98%, Sigma-Aldrich) was prepared in chloroform (Fisher Scientific). The ODT solution (10 mL) was added to a 100 mL beaker along with 50 mL of 0.05 mg/mL AuNP (diameter of either 10.9 ± 0.8, 19.9 ± 1.5, 40 ± 3, or 83 ± 7 nm by TEM) in 2 mM sodium citrate solution (nanoComposix). The beaker contents were sonicated for 3 min using a Branson Sonifer 250 probe sonicator (250 W, 20 kHz). After the aqueous and chloroform phases were separated, the ODT-AuNPs in chloroform were recovered into vials and sonicated in a bath sonicator until the AuNPs were well-dispersed (∼30 min). These AuNP solutions sat undisturbed overnight to allow the remaining ODT ligands to continue to bind to the AuNP surfaces. The excess ligand was removed by pelleting the AuNPs in an Eppendorf Centrifuge 5840 at a speed of 10,000 rpm for 10 min and removing the supernatant. The AuNPs were then dispersed in a 9:1 toluene (Sigma-Aldrich, anhydrous, 99.8%):chloroform solution to an absorbance of ∼1.5 at AuNP λ_max_, as measured using a VWR PV4 visible spectrophotometer.

### Nanoparticle Characterization

Transmission electron microscope (TEM) studies of the AuNPs after the ligand exchange were performed by depositing 5 μL of the AuNP solutions onto carbon type-B, 300 mesh, copper TEM grids from Ted Pella and collecting images using a JEOL 2011 TEM operated at 200 kV. ODT-AuNP size distributions were determined by first measuring the diameters of at least 170 particles per sample in ImageJ and then fitting the diameter data to Gaussian distributions. The errors reported with the distributions are ±1 standard deviation.

### Peptoid Nanosheet Formation

The peptoids used in this study were custom synthesized by Cambridge Research Biochemicals (purity >80% by HPLC, Figure S1). All water used in solution preparation was obtained from a Millipore Synergy UV Water Purification System and had a resistivity of 18.2 MΩ·cm. A stock 2 mM peptoid solution was prepared in 2:1 dimethyl sulfoxide (Sigma-Aldrich):water to prevent nanosheet formation at the air–water interface. The working peptoid solution was prepared in a 1-dram vial by diluting the peptoid stock to a concentration of 20 μM in a pH 8, 10 mM Trizma base (Sigma-Aldrich) solution to a final volume of 500 μL. On top of the peptoid solution was added 500 μL of either toluene (for forming bare nanosheets) or ODT-AuNP solution (Abs. at λ_max_ = 1.5). Peptoid nanosheets were prepared by holding the vials in a horizontal position for 2 min to allow for assembly of the composite peptoid-AuNP monolayer. The vials were then rotated to a vertical position to reduce the interfacial area and allow for monolayer collapse. The vials were then immediately returned to the horizontal position. This process of allowing monolayer assembly for 2 min and subsequent compression by rotating the vial was repeated a total of 10 times. After rotating, the organic layer with remaining AuNPs was manually removed from the vials by using a glass pipet.

### Peptoid Nanosheet Characterization

The ODT-AuNP peptoid nanosheets in solution were imaged using an Olympus BX51 optical microscope, in which 10 μL of the peptoid solutions was deposited onto thermal silicon oxide wafers (300 nm SiO_2_ layer on Si(100), MTI Corp.) that had been washed with acetone and subsequently cleaned in a Harrick Plasma Cleaner. For AFM and SEM characterization, nanosheets were purified from free peptoid and buffer via pelleting in a Corning MiniCentrifuge for ∼5 min, removing the supernatant, and redispersing in 400 μL of pure water. The purified nanosheet solutions (10 μL) were deposited on clean thermal silicon oxide wafers and dried in a vacuum desiccator. AFM images were collected using a VEECO Dimension V AFM under ambient conditions in tapping mode using silicon AFM probes with aluminum reflective coatings from Ted Pella (resonant frequency of 150 kHz and force constant of 5 N/m). The AFM images were processed in Gwyddion. For nanosheets prepared in the absence of AuNPs, the average nanosheet thickness was calculated from height profiles of 5 different nanosheets. For nanosheets formed with ODT-AuNPs, average thicknesses for the bare regions of the nanosheet and bare substrate were determined from height profiles of 20 different nanosheet images taken at a scan size of 5 μm × 5 μm. Average thicknesses due to the ODT-AuNPs within the nanosheets were calculated from 10 different nanosheet images per sample taken at a scan size of 5 μm × 5 μm. Here, masks were superimposed only on regions of the nanosheets in which AuNPs were present. The average height of the masked region was calculated for each nanosheet, and these heights were averaged for each sample. Regions of the nanosheets with folds or wrinkles were not included in the masks. SEM images were collected on a Thermo-Fisher Versa 3D Dual Beam System.

## Results and Discussion

Peptoid nanosheets in the absence of AuNPs were successfully prepared at the toluene–aqueous interface, as shown in Figure 2. The light microscope image of a nanosheet dried on a thermal silicon oxide wafer (Figure 2A) shows a flat material with straight edges, consistent with peptoid nanosheets formed via the monolayer collapse mechanism.^29^ Some wrinkles and folds are also evident. These features are also observed in the SEM image of this nanosheet (Figure 2B). AFM data (Figure 2C) were collected to measure the average thickness of the peptoid nanosheet in the absence of AuNPs. A representative height profile of a nanosheet edge is shown in Figure 2D. Based on analysis of height profiles from five different nanosheets, the average thickness of the peptoid nanosheet was calculated to be 2.5 ± 0.4 nm, which is consistent with previous AFM studies of peptoid nanosheets.^33,44^ Overall, the data in Figure 2 demonstrate that the biphasic monolayer adsorption assembly mechanism can produce bare peptoid nanosheets that are relatively flat and thin.

**Figure 2:**
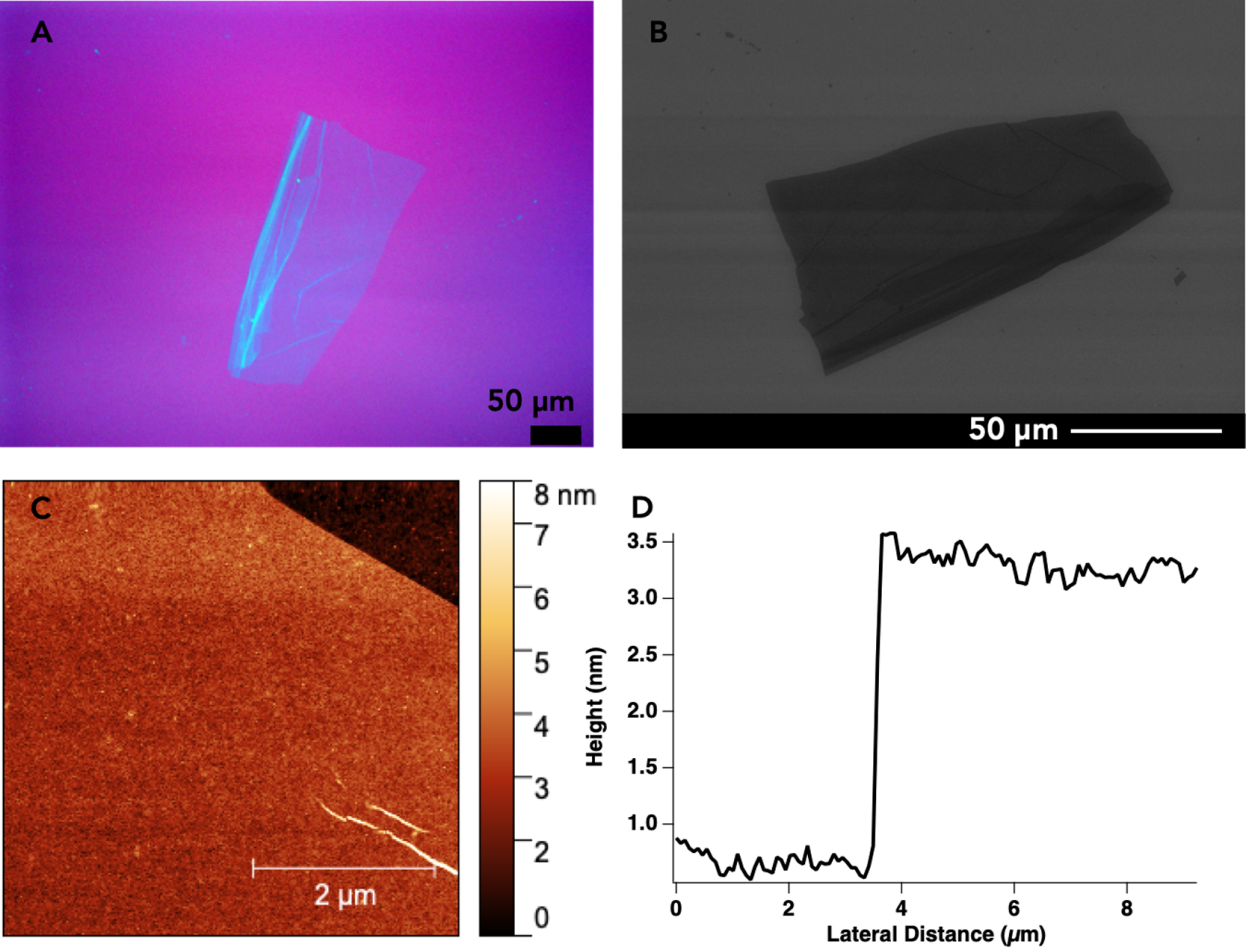
Characterization of peptoid nanosheets formed via the biphasic monolayer adsorption assembly mechanism in the absence of AuNPs. (A) Light microscope image of a dry nanosheet deposited on a thermal silicon oxide wafer. (B) SEM image of the nanosheet in (A). (C) AFM image of a dry nanosheet edge taken at a scan size of 5 × 5 μm. (D) Representative height profile of a nanosheet’s edge used to calculate the average thickness of the bare nanosheets.

To prepare the AuNP nanosheets via the biphasic monolayer assembly mechanism, the AuNPs must be soluble in toluene solution so that they can be incorporated into the peptoid nanosheet’s hydrophobic interior. Therefore, AuNPs require functionalization with a hydrophobic ligand. Aqueous solutions of citrate-AuNPs with diameters of 10, 20, 40, and 80 nm were sonicated with chloroform solutions of ODT. The AuNPs were successfully functionalized with ODT ligands, as demonstrated by their transfer from the aqueous phase to chloroform (Figure S2). TGA-FTIR (Figures S3–S8) and NMR (Figures S9–S10) studies confirmed that the ligands were bound to the AuNP surfaces. The visible spectra of the AuNPs before and after functionalization and phase transfer (Figure S11) demonstrated a red-shifting of the plasmonic peaks, which is consistent with changing the AuNP environment from water to toluene. The diameters of the AuNPs were measured after functionalization with ODT by TEM (Figure 3) to know the sizes of particles that could be incorporated into the peptoid nanosheets.

**Figure 3:**
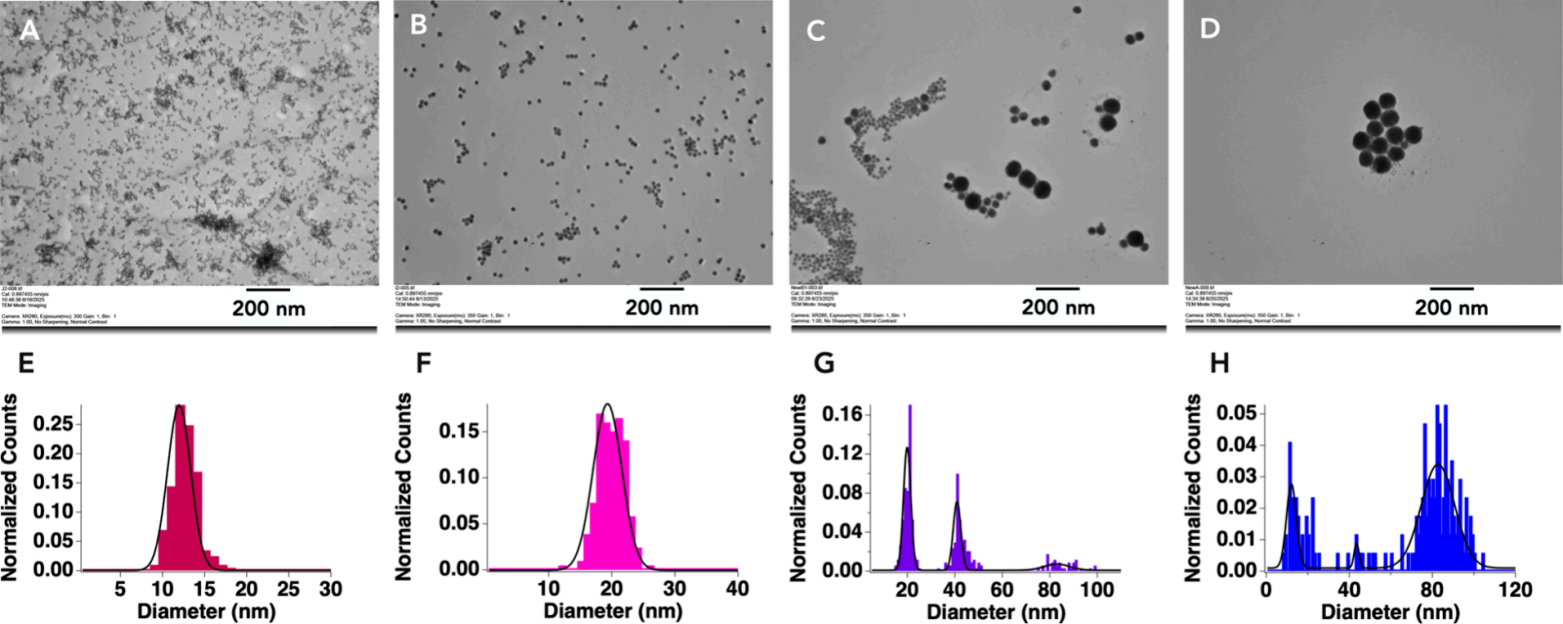
TEM data of the ODT-AuNPs prepared from the 10 (A), 20 (B), 40 (C), and 80 nm (D) citrate-AuNP samples. Average diameters calculated from the size distributions of each ODT-AuNP sample are 12 ± 1 nm (E), 19 ± 2 nm (F), 40 ± 20 nm (G), and 70 ± 30 nm (H). All images were taken at a magnification of 80,000×.

The size distributions from TEM data (Figure 3E–H) show single populations for the ODT-AuNP samples prepared with 10 nm (Figure 3A and E, 12 ± 1 nm) and 20 nm (Figure 3B and F, 19 ± 2 nm) citrate-AuNPs, while three size populations are seen in the ODT-AuNP samples prepared from 40 nm (Figure 3C and G, 20 ± 2, 41 ± 2, 83 ± 5 nm) and 80 nm (Figure 3D and H, 12.2 ± 0.4, 44 ± 1, 83 ± 11 nm) citrate-AuNPs. Previous work has established that sonicating AuNPs with alkanethiols can lead to competing processes of particle fusion, which increases the AuNPs’ diameters, and digestive ripening, which decreases the AuNPs’ diameters.^45^ Particle fusion is caused by the elevated temperatures that are brought about by sonication, and digestive ripening occurs due to the presence of the free alkanethiol ligands that etch the particles’ surfaces. It is thus likely that the 40 nm sample experienced digestive ripening and particle fusion during the ligand exchange process, while the 80 nm sample underwent digestive ripening. These polydisperse samples are of interest, as they can demonstrate if a single nanosheet can accommodate differently sized cargo. The samples are distinct, however, because the 40 nm sample contains a majority of particles with diameters near 20 and 40 nm (Figure 3G), while the 70 nm sample contains a majority of particles with diameters near 12 and 83 nm (Figure 3H). Average AuNP diameters were calculated for each sample from the TEM data: 12 ± 1 nm (Figure 3E), 19 ± 2 nm (Figure 3F), 40 ± 20 nm (Figure 3G), and 70 ± 30 nm (Figure 3H). For the rest of this work, the different samples of the ODT-AuNP will be referred to by their average diameter.

The ODT-AuNP samples were prepared for nanosheet synthesis by dispersing in 9:1 toluene:chloroform solution at an absorbance near 1.5 at λ_max_ (Figure S11). Concentrations were estimated to be near 0.1 mg/mL for all samples, which were calculated based on the work of Haiss et al.^46^ (see the Supporting Information). The ODT-AuNPs were added to a vial with the peptoid solution, and nanosheets were formed by gently rocking the vials from a horizontal to vertical orientation. Previous studies have shown that this vial rocking method results in monolayer compression and collapse, leading to the formation of peptoid nanosheets.^26^ Figure 4 shows the vials before (A) and after (B) rotation. There is a pink-purple color in the aqueous phases for all AuNP diameter samples after rotation (Figure 4B) that was not present before vial rotation (Figure 4A). The color is well dispersed throughout the aqueous phase, which suggests the formation of peptoid nanosheets with ODT-AuNPs embedded in the hydrophobic interiors and the hydrophilic groups of the peptoid exposed on the nanosheet surfaces.

**Figure 4:**
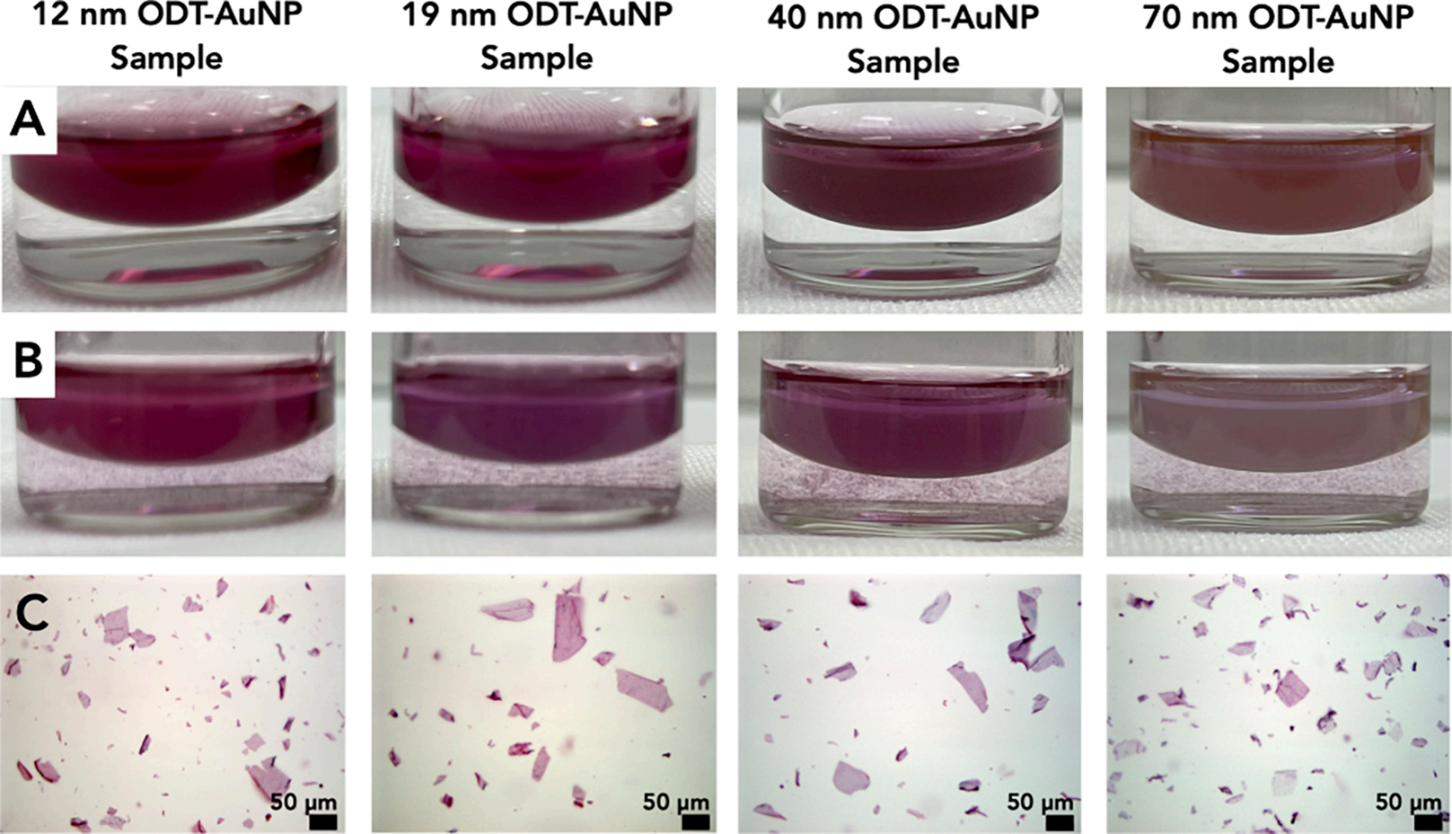
Formation of peptoid nanosheets with the 12, 19, 40, and 70 nm ODT-AuNP samples. Photographs of vials containing 20 μM peptoid in the aqueous phase and either the 12, 19, 40, or 70 nm ODT-AuNP samples in 9:1 toluene:chloroform before (A) and after vial rotation (B). (C) Light microscope images of peptoid nanosheets formed with either the 12, 19, 40, or 70 nm ODT-AuNP samples.

The insertion of the ODT-AuNPs into the nanosheets’ interiors is supported by optical microscope images of the aqueous phases after vial rotation (Figure 4C). For nanosheets prepared with the 12, 19, and 40 nm ODT-AuNP samples, flat pink/purple sheets are observed in which the colors are mostly uniform throughout these nanosheets. For nanosheets prepared with the 70 nm ODT-AuNP sample, some sheets are darker in color than others, suggesting that some sheets contain a higher density of ODT-AuNPs than others. To determine the arrangement of the AuNPs within the peptoid nanosheets, AFM (Figure 5) and SEM (Figure 6) images were collected.

**Figure 5:**
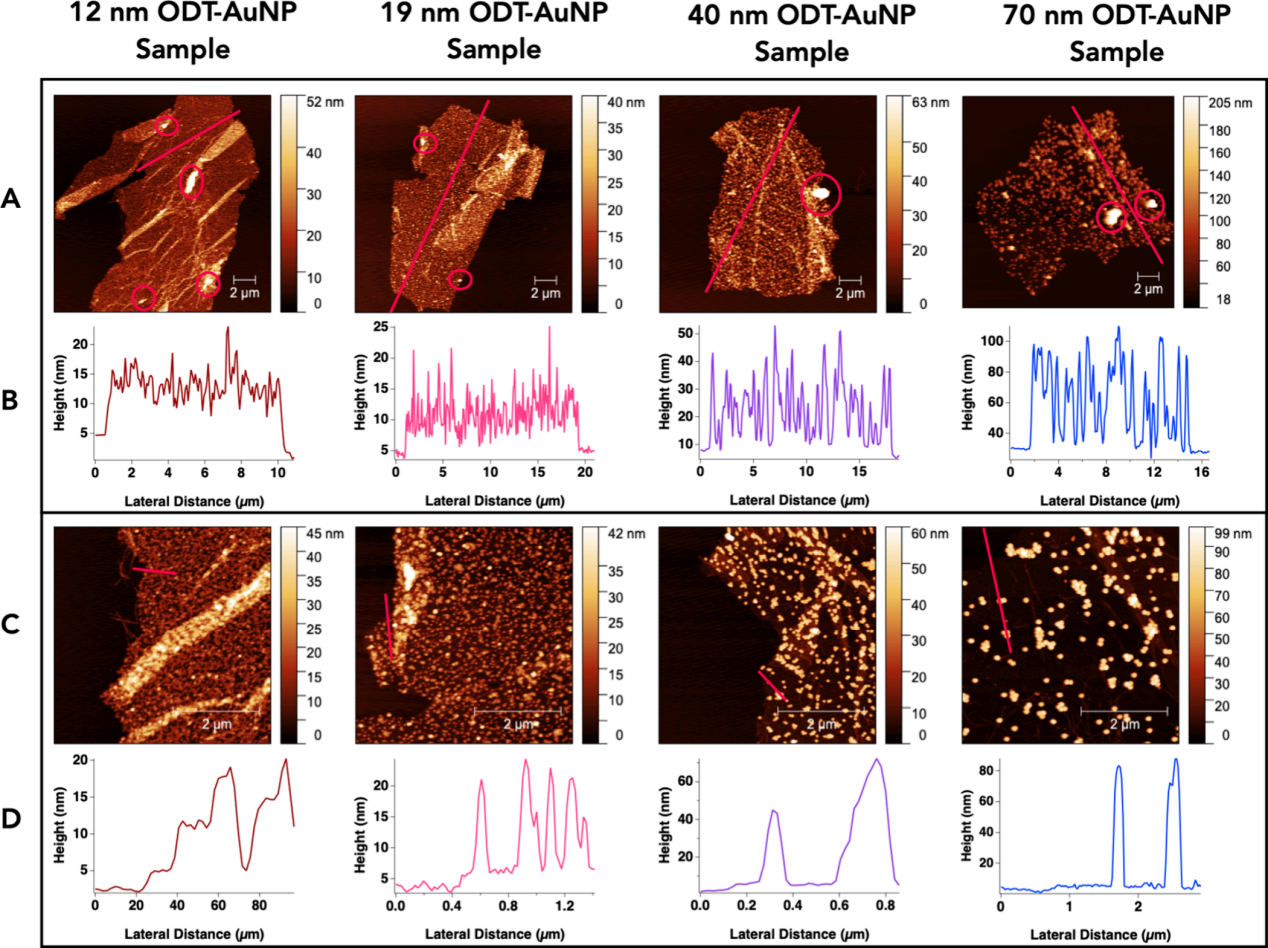
Representative AFM data of peptoid nanosheets prepared with either 12, 19, 40, or 70 nm ODT-AuNP samples. The images in (A) display nanosheets taken at a scan size of 20 μm × 20 μm, in which likely agglomerates are circled. Height profiles were obtained along the red lines in (A), which are shown in the graphs in (B). The images in (C) display sections of the nanosheets in (A) taken at a scan size of 5 μm × 5 μm. Height profiles were obtained along the red lines in (C), which are shown in the graphs in (D).

**Figure 6:**
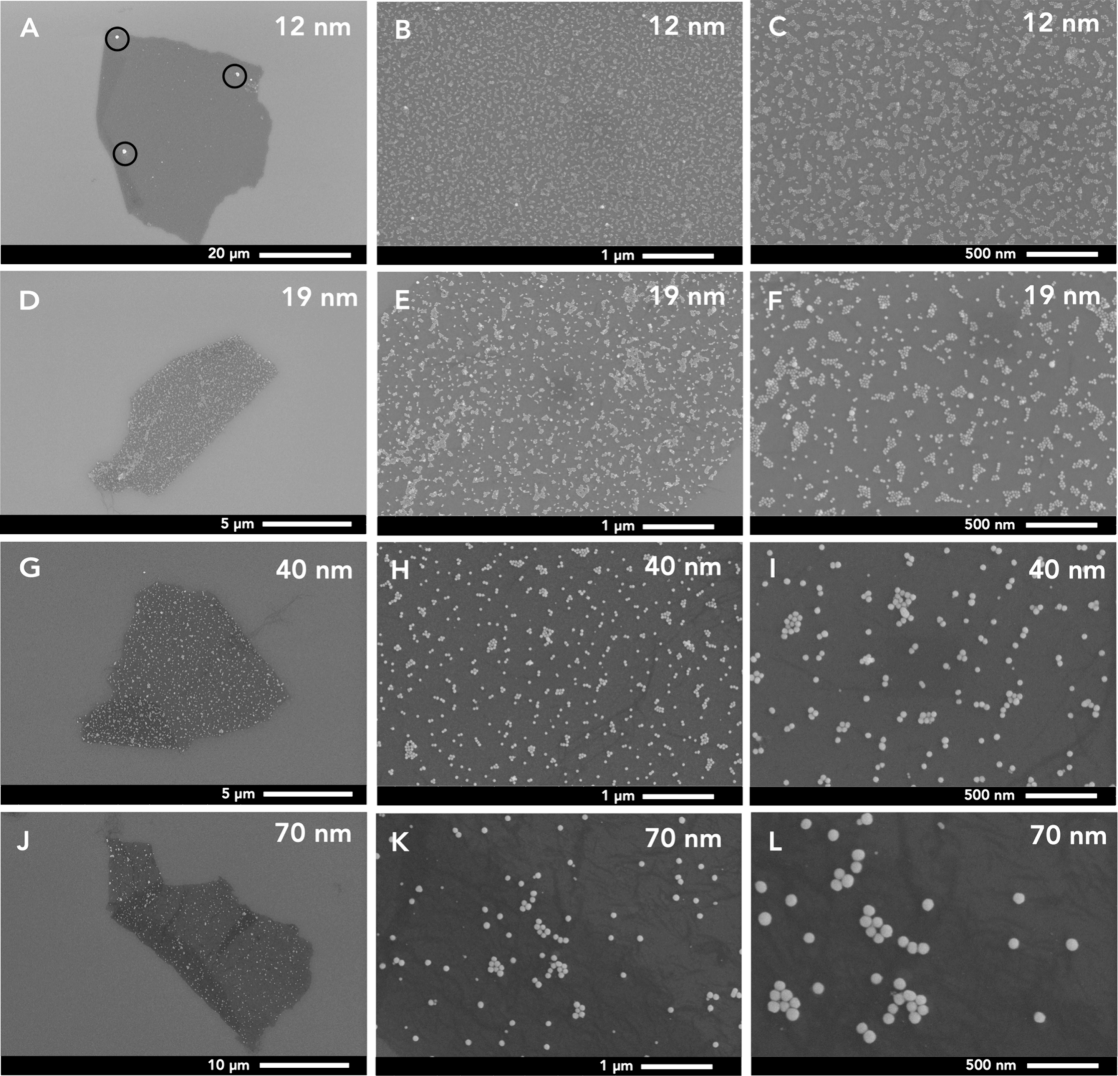
SEM images of peptoid nanosheets formed with the 12 (A–C), 19 (D–F), 40 (G–I), and 70 nm (J–L) ODT-AuNP samples. Images were collected at different magnifications: 2500× (A), 5000× (J), 10,000× (D, G), 40,000× (B, E, H, K), and 80,000× (C, F, I, L). ODT-AuNP agglomerates are circled in A.

The AFM images of the nanosheets were used to determine the approximate number of AuNP layers embedded in the nanosheets. Height profiles were collected along the lines superimposed on the AFM images in Figure 5A. These profiles (Figure 5B) display heights that generally do not exceed more than one AuNP diameter, which is consistent with one AuNP layer. However, there are some large white features in the AFM images in Figure 5A with heights of 100–300 nm. These features are likely agglomerated AuNPs in the nanosheet interior. Other features observed in the nanosheets are wrinkles and folds, which are thicker than the flatter regions of the nanosheets.

AFM images of the nanosheets in Figure 4A were collected at a smaller scan size to better observe the packing of the AuNPs in the sheets (Figure 5C). These AFM images also reveal regions of the nanosheets where the ODT-AuNP packing density is low. These images allow for determining the thickness of the bare nanosheet, in relation to the bare substrate. Height profiles in Figure 5D show that the average nanosheet thickness in the absence of AuNPs is 2.6 ± 0.5 nm, which is consistent with the average thickness of the nanosheet with an empty interior (Figure 2C and D). Thicknesses due to the ODT-AuNPs within the nanosheets were also determined by measuring the heights at which AuNPs are present in the nanosheets. Upon subtracting the heights due to the bare nanosheet and bare substrate, these average thicknesses were determined to be 10 ± 3 nm for the 12 nm sample, 17 ± 3 for the 19 nm sample, 34 ± 3 for the 40 nm sample, and 67 ± 8 nm for the 70 nm sample. As was seen in the height profiles in Figure 5B, these calculated AuNP thicknesses are consistent with an average of one layer of ODT-AuNPs within the nanosheets.

The arrangement of AuNPs within the nanosheets was further observed in representative SEM images, in which single particles appear as bright, well-defined spheres (Figure 6). Images taken at a higher magnification (300,000×) are shown in Figure S12, which display features consistent with electron beam damage of organic material in proximity to the gold nanoparticles. We discuss these features in the Supporting Information (Figures S12–S14). TEM images were also collected but did not display a contrast between the peptoid nanosheet and the carbon support on the TEM grid (Figure S15).

The extent of packing of the AuNPs within the peptoid nanosheets is indicative of adsorption of the ODT-AuNP to the peptoid monolayer intermediate. The SEM images show areas within the nanosheets where the ODT-AuNPs tend to adopt a single layer of tightly packed arrangements, which suggests that the assembly of the ODT-AuNP to the peptoid monolayer is driven not only by interactions between the peptoid hydrophobic groups and the hydrophobic ODT groups on the AuNP surfaces but also by AuNP–AuNP interactions. As seen Figure 6B, E, H, and K, the packing density also decreases with increasing AuNP diameter. This result is consistent with the AFM data. Nanoparticle assembly at fluid interfaces is influenced in part by the bulk nanoparticle concentration and the nanoparticle diffusion coefficient.^47,48^ Our previous work has shown that increasing the bulk concentration of 5 nm DDT-AuNPs increases their adsorption to the peptoid monolayer hydrophobic face.^34^ While the estimated mass concentrations of the ODT-AuNP samples are about the same (0.1 mg/mL), the estimated molar concentrations for the 12 nm (8.59 nM), 19 nm (1.75 nM), 40 nm (0.160 nM), and 70 nm (0.0270 nM) of the ODT-AuNP samples are quite different (Table S2). Experimental limitations did not make it feasible to obtain higher concentrations for the 40 and 70 nm ODT-AuNP samples (see the SI); thus, we performed the experiments at a constant mass concentration of ∼0.1 wt %. The lower molar concentrations for the larger AuNPs likely limited their extent of adsorption to the peptoid monolayer. Moreover, AuNPs with larger diameters have smaller diffusion coefficients than AuNPs with smaller diameters, so the adsorption of larger AuNPs to the peptoid monolayer is slower than it is for smaller AuNPs. The packing density thus cannot be accurately compared between the different samples. Future work in our lab will focus on controlling the packing density of the ODT-AuNPs within the peptoid nanosheets.

The SEM images also reveal the heterogeneity of the AuNP sizes packed within the individual nanosheets. For nanosheets prepared with 12 nm ODT-AuNPs (Figure 6A), large white features with diameters of ∼0.1–1 μm are seen that are indicative of agglomerated AuNPs in the nanosheet. A representative SEM image of agglomerated ODT-AuNPs is shown in Figure S16. For nanosheets prepared with 40 nm (Figure 6I) and 70 nm ODT-AuNPs (Figure 6L), a range of particle sizes are observed in the SEM images of the nanosheets, as is expected from the digestive ripening of the 40 and 80 nm citrate-AuNPs that occurred during the ligand exchange. These SEM images show that a single nanosheet can accommodate a variety of differently sized cargo, which reveals the versatility of this freely floating nanomaterial.

## Conclusions

We have demonstrated here that we can successfully functionalize 10, 20, 40, and 80 nm citrate-AuNPs with hydrophobic ODT ligands using a phase transfer process that is facilitated by sonication. The ODT-AuNPs can be dispersed in a toluene solution and incorporated into the nanosheet interior by gently rocking vials containing the ODT-AuNP and peptoid solutions. We can thus embed hydrophobic cargo into the peptoid nanosheet hydrophobic core with diameters between ∼10 and ∼80 nm and potentially up to hundreds of nanometers. Moreover, a single nanosheet can accommodate a variety of differently sized cargos, demonstrating the adaptability of the material. AFM and SEM data reveal that the nanosheets encapsulate on average one patchy layer of ODT-AuNPs. Both isolated ODT-AuNPs and regions of tightly packed ODT-AuNP arrangements are observed. The intermolecular interactions that hold together the single peptoid chains at the oil–water interface are significant enough to create mechanically strong monolayers capable of encapsulating cargo that is relatively large compared to the monolayer thickness. Moreover, the intermolecular interactions between the peptoid monolayer hydrophobic faces and the hydrophobic ODT ligands are robust enough to hold the material together.

Our results have revealed how powerful the biphasic adsorption assembly mechanism is. Peptoid monolayer assembly at collapse at the oil–water interface allows for functionalizing not only the nanosheet hydrophilic exterior but also the hydrophobic interior with a variety of differently sized inorganic cargos. One can imagine using peptoid nanosheets as multifunctional platforms that freely float in aqueous solutions. Here, the nanosheet exterior could be designed to interact with species in solution, and the nanosheet interior could be loaded with inorganic nanoparticles that impart useful optical, electronic, magnetic, catalytic, or reactive properties. Overall, this work has shown that the peptoid nanosheet is a customizable and robust material that can serve as a platform for the assembly of functional hybrid 2D nanomaterials with potential applications in drug delivery, catalysis, and sensing.

## Supplementary Material



## Notes

Peptoid characterization by HPLC, images of the gold nanoparticles before and after functionalization with octadecanethiol; TGA-FTIR, ^1^H NMR, 2D DOSY NMR, and visible spectroscopy results; supporting electron microscopy data (PDF)
