# Supplementary material for: Incorporating Gold Nanoparticles with Varying Diameters into Freely Floating Nanosheets via a Biphasic Monolayer Adsorption Assembly Mechanism
Source: Langmuir. 2025 Dec 10;41(50):33892–900. doi: 10.1021/acs.langmuir.5c04350 (PMC12750992; doi:10.1021/acs.langmuir.5c04350)
Supplement: Supplementary file 1 [file la5c04350_si_001.pdf]

Supporting Information

Incorporating Gold Nanoparticles with Varying Diameters into Freely Floating Nanosheets via a Biphasic Monolayer Adsorption Assembly Mechanism

Ellen J. Robertson[a]*, Chao Yang[b], Antonia Sofia Soto Carrillo[a], C'Lannye James[a], and Christopher B. Whitehead[a]

a. Chemistry Department, Union College, 807 Union St., Schenectady, New York 12308, United States

b. Materials Science and Engineering Department, Rensselaer Polytechnic Institute, Troy, New York 12180, United States

*email: roberte2@union.edu

Table of Contents

1. Peptoid Characterization S2
2. AuNP Ligand Exchange and Characterization S3
    a. AuNP Phase Transfer S3
    b. TGA-FTIR Studies S4
    c. NMR Studies S11
    d. AuNP Visible Spectroscopy Data S14
3. Supporting Electron Microscopy Data S16
    a. Electron Beam Damage to the Nanosheets S16
    b. TEM Images of the Gold Peptoid Nanosheets S19
    c. Agglomerated AuNPs S21
4. References S22

1. Peptoid Characterization

The peptoid used in these studies was custom synthesized by Cambridge Research Biochemicals (crb). The HPLC trace in Figure S1 was supplied by crb.

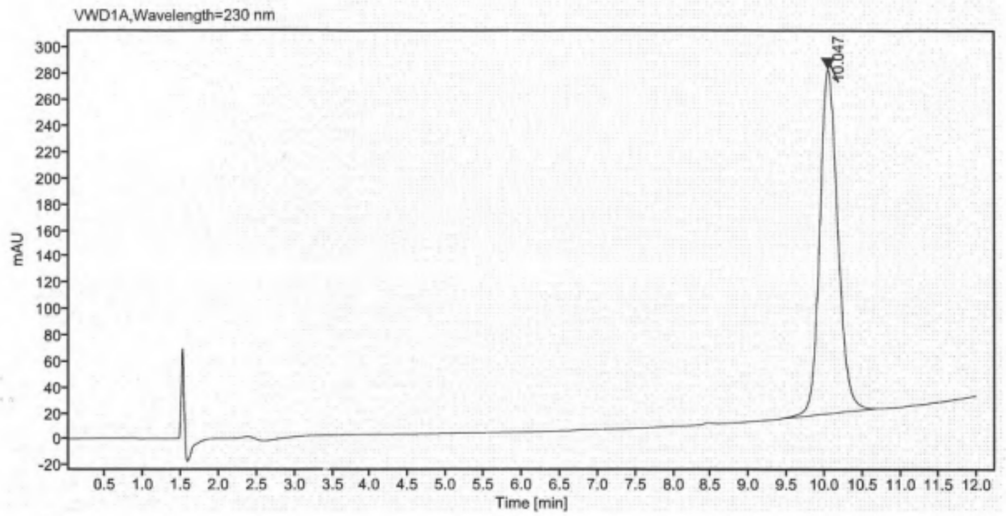

Signal: VWD1A,Wavelength=230 nm

RT [min]	Width [min]	Area	Height	Area%
10.047	1.3777	4445.2478	263.8628	100.0000
	**Sum**	**4445.2478**		

Figure S1. HPLC analysis report for the peptoid used in these studies. The theoretical monoisotopic mass of this peptoid is 3400.2. According to the Certificate of Analysis from crb, a mass of 1134.4 (1/3$[M+3H]^{3+}$) was found via mass spectrometry analysis.

2. AuNP Ligand Exchange and Characterization

a. AuNP Phase Transfer

The functionalization of hydrophilic gold nanoparticles (AuNPs) with different hydrophobic ligands like alkanethiols and alkane amines has been accomplished via phase transfer processes, in which the AuNPs transfer from the aqueous phase to the organic phase upon binding the hydrophobic ligands.[1–8] A ligand exchange was thus performed by mixing an aqueous solution of citrate-AuNPs with a chloroform solution of octadecanethiol (ODT). An ODT concentration of 0.4 mM was used. This concentration is 10 x the molar amount needed to achieve a ligand packing density of 5 ligands/nm^2, which has been reported as the approximate maximum packing of alkanethiol ligands on AuNP surfaces.[9] A probe sonicator was used to induce the mixing of the organic phase containing the ligand and the aqueous phase containing the AuNPs. After sonication, the pink color of the AuNPs in the aqueous phase faded, while a dark purple color appeared in the chloroform phase (Figure S2). This phase transfer was evidence of a successful functionalization of the AuNPs with the hydrophobic ODT ligand.

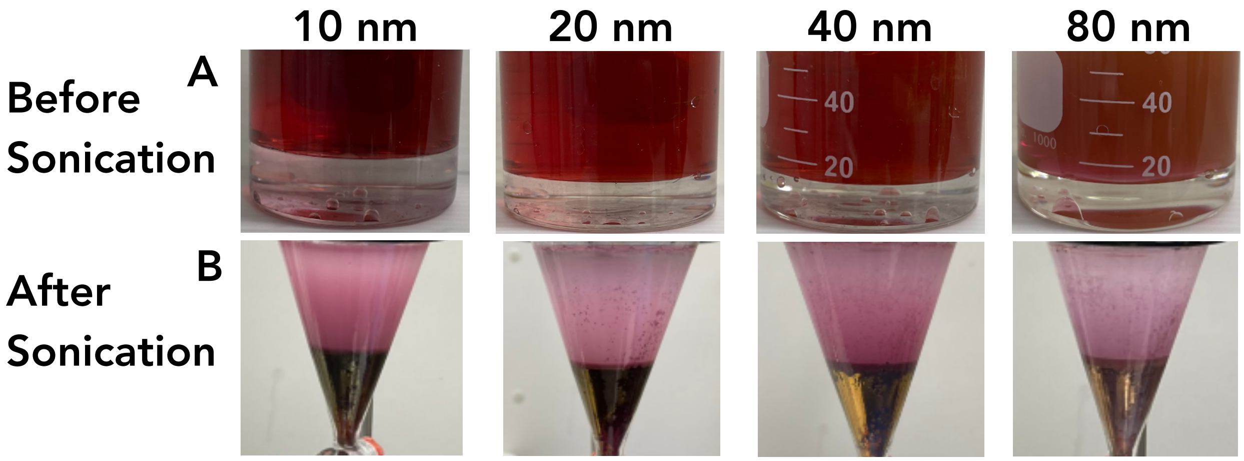

Figure S2. Photographs demonstrating the transfer of AuNPs from the aqueous phase to the chloroform phase upon functionalization with hydrophobic ODT ligands. (A) Beakers containing 50 mL of 0.05 mg/mL AuNPs with diameters near 10, 20, 40, and 80 nm in citrate buffer (top layers) and 10 mL of 0.4 mM octadecanethiol in chloroform (bottom layers). (B) After sonicating for 3 minutes, the contents of the beakers in (A) were transferred into to 150 mL separatory funnels where the chloroform and aqueous phases were allowed to separate. Much of the pink color from the aqueous phases transferred to chloroform after the ligand exchange.

b. TGA-FTIR Studies

The ODT-AuNPs were characterized using a hyphenated thermal gravimetric analyzer-Fourier transform infrared spectrometer (TGA-FTIR) system from PerkinElmer (TGA 8000 and Spectrum 3 MIR/NIR FTIR). Briefly, purified ODT-AuNPs were dispersed in a minimal amount of chloroform. The chloroform-AuNP solutions were pipetted into pre-tared ceramic TGA sample pans, and the chloroform was allowed to evaporate. Typical initial AuNP sample masses as measured by the TGA balance were ~1-5 mg. In the TGA-FTIR experiments, the AuNPs were heated from 30 °C to 90 °C, holding at 90 °C for 5 mins to remove any excess solvent present. Then, the samples were heated to 700 °C at a rate of 20 °C/min under nitrogen gas flowing at a rate of 20 mL/min. A pump carried any evolved gases from the TGA to the FTIR through transfer lines that were heated to 270 °C. IR spectra (4000 cm^{-1} – 600 cm^{-1}, 8 cm^{-1} resolution) were collected continuously throughout the TGA experiment. Each spectrum collected was an average of 2 scans. TGA-FTIR data were also collected of pure ODT (Figure S3) and sodium citrate dihydrate (Figure S4) samples. These are qualitative studies, as the polydispersity of the ODT-AuNP samples makes it challenging to calculate the number of ligands per particle. Instead, we focus on characterizing different ligand environments on the particle surfaces.

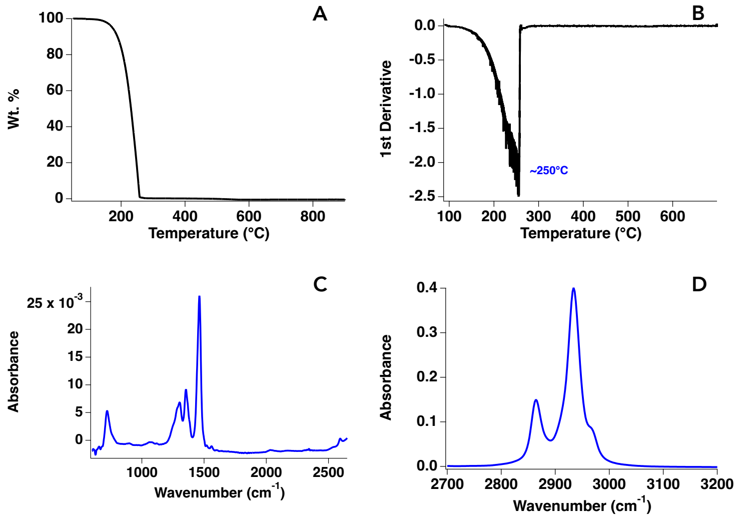

Figure S3. Representative TGA-FTIR data for ODT. The thermogram (A) and its first derivative (B), along with FTIR spectra of the evolved gases in the low (C) and high (D) frequency regions. A single mass loss event is seen near 250 °C. Significant peaks were not observed outside of the regions presented in C and D.

Figure S3A shows the thermogram for the pure ODT ligand. A single mass loss event is seen that occurs between ~150 °C and 260 °C, with first derivative of the thermogram (Figure S3B) showing a minimum near 205 °C. The FTIR spectrum of the evolved gases (Figures S3C-D) is consistent with the literature spectrum of the free octadecanethiol ligand in the gas phase.[10] Here, peaks are seen near 2800 cm^{-1} – 3000 cm^{-1} (methyl and methylene stretching modes)[11],

2594 cm^{-1} (S-H stretching mode)[11], 1462 cm^{-1} (methylene scissoring mode)[11], 1354 cm^{-1} & 1304 cm^{-1} (methyl bending modes)[11], and 720 cm^{-1} (C-S stretching mode)[11]. These data suggest that the free ODT does not decompose but rather vaporizes as a whole molecule near 250 °C. Our result is consistent with TGA-gas chromatography-mass spectrometry (TGA-GC-MS) studies of pure dodecanethiol (DDT), which was shown to vaporize rather than decompose near 150 °C.[9]

The thermogram of sodium citrate dihydrate (Figure S4A) shows three mass loss events between 90 °C and 700 °C.

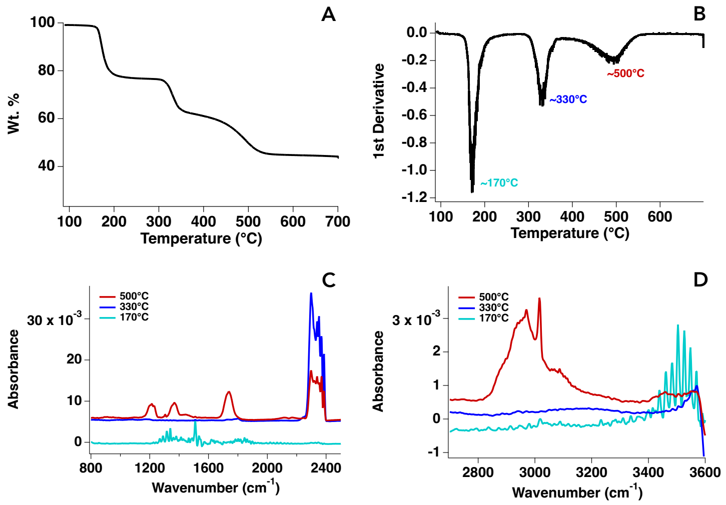

Figure S4. Representative TGA-FTIR data for sodium citrate dihydrate. The thermogram (A) and its first derivative (B), along with FTIR spectra of the evolved gases in the low (C) and high (D) frequency regions. Three mass loss events are seen near 170 °C (teal trace), 330 °C (blue trace), and 500 °C (red trace). Significant peaks were not observed outside of the regions presented in C and D. The spectra are offset for clarity.

The first derivative of the thermogram (Figure S4B) shows that these mass loss events occur near 170 °C, 330 °C, and 500 °C. As seen in the FTIR spectrum of the evolved gas at 170 °C (Figure S4C-D, teal trace), peaks appear near 3500 cm^{-1} (water OH stretching mode)[11] and 1200 cm^{-1} – 1500 cm^{-1} (water OH bending mode)[11], consistent with the dehydration of citrate. At 330 °C (blue trace), the FTIR spectrum is dominated by a peak near 2300 cm^{-1} (carbon dioxide C=O stretching mode)[10], suggesting partial decarboxylation. At 500 °C (red trace), further decarboxylation is apparent from the peak near 2300 cm^{-1}. Additional relevant peaks appear near 1214 cm^{-1} (C-O stretching mode)[11], 1738 cm^{-1} (C=O stretching mode)[11], and a broad peak from 2800 cm^{-1} – 3200 cm^{-1} (carboxylic acid OH stretching modes)[11]. These peaks are consistent with the decomposition of the material remaining at 500 °C into small carboxylic acid containing molecules. Overall, the data are consistent with previous studies on the pyrolysis of sodium citrate.[12]

Representative TGA-FTIR data for 12, 19, 40, and 70 nm ODT-AuNP samples are shown in Figures S5-S8. At least three TGA-FTIR data sets were collected for each type of ODT-AuNP, and all data showed similar features. Thus, the TGA-FTIR data for all ODT-AuNP samples will be discussed together.

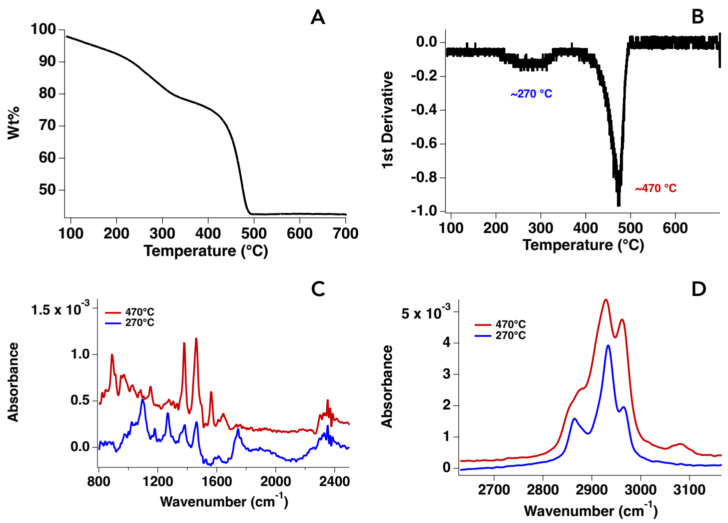

Figure S5. Representative TGA-FTIR data for the 12 nm ODT-AuNP sample. The thermogram (A) and its first derivative (B), along with FTIR spectra of the evolved gases in the low (C) and high (D) frequency regions. Two mass loss events are seen near 270 °C (blue trace) and 470 °C (red trace). Significant peaks were not observed outside of the regions presented in C and D. The spectra are offset for clarity.

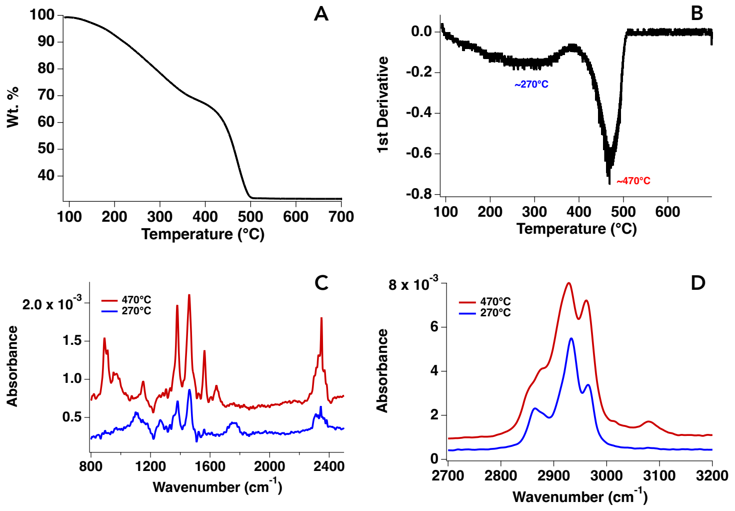

Figure S6. Representative TGA-FTIR data for 19 nm ODT-AuNPs. The thermogram (A) and its first derivative (B), along with FTIR spectra of the evolved gases in the low (C) and high (D) frequency regions. Two mass loss events are seen near 270 °C (blue trace) and 470 °C (red

trace). Significant peaks were not observed outside of the regions presented in C and D. The spectra are offset for clarity.

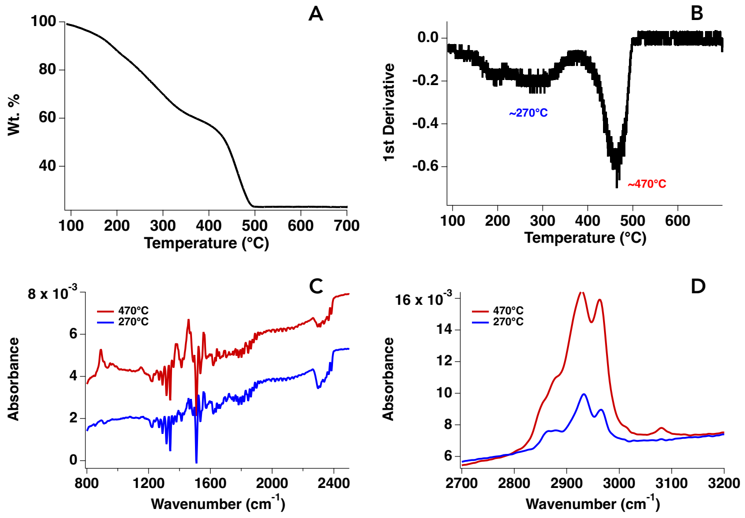

Figure S7. Representative TGA-FTIR data for the 40 nm ODT-AuNP sample. The thermogram (A) and its first derivative (B), along with FTIR spectra of the evolved gases in the low (C) and high (D) frequency regions. Two mass loss events are seen near 270 °C (blue trace) and 470 °C (red trace). Significant peaks were not observed outside of the regions presented in C and D. The spectra are offset for clarity.

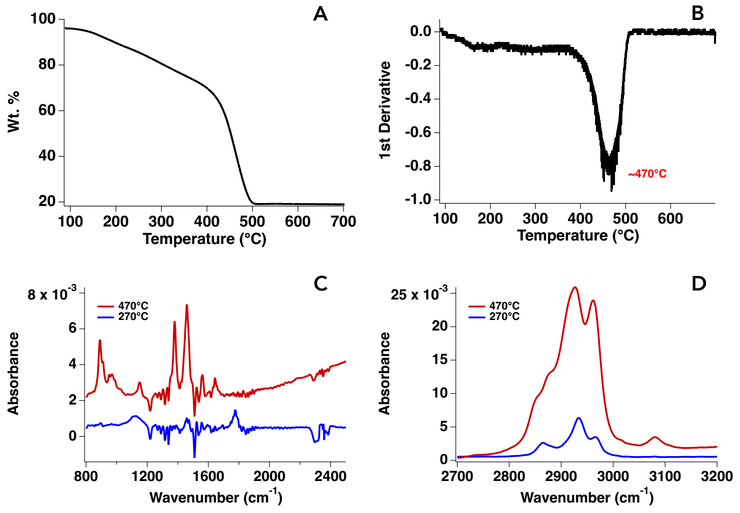

Figure S8. Representative TGA-FTIR data for the 70 nm ODT-AuNP sample. The thermogram (A) and its first derivative (B), along with FTIR spectra of the evolved gases in the low (C) and high (D) frequency regions. A gradual mass loss is observed between 100 and 400 °C (blue trace, 270 °C), with a steeper mass loss occurring near 470 °C (red trace). Significant peaks were not observed outside of the regions presented in C and D. The spectra are offset for clarity.

The thermograms and their first derivatives for the 12 nm (Figure S5A, B), 19 nm (Figure S6A, B), and 40 nm (Figure S7A, B) ODT-AuNPs show two clear mass loss events near 270 °C and 470 °C. While the mass loss event near 470 °C is also present in the 70 nm ODT-AuNP TGA data (Figure S8A, B), there is not a well-defined mass loss event near 270 °C. However, the mass does gradually decrease between ~100 °C and 400 °C, suggesting gas evolution at these lower temperatures that is distinct from the mass loss event at the higher 470 °C temperature. The gases produced during these different temperature regimes can be elucidated from the FTIR data at 270 °C (blue traces in Figures S5-8C and D) and 470 °C (red traces in Figures S5-8C and D). At 270 °C, peaks appear between 2800 cm^{-1} – 3000 cm^{-1} (methyl and methylene stretching modes)[11], 1386 cm^{-1} (methyl bending mode)[11], and 1466 cm^{-1} (methylene scissoring mode)[11] that are consistent with a linear aliphatic chain such as an alkane or octadecanethiol. The decomposition of octadecanethiol ligands to an alkane is consistent with TGA-GC-MS studies of DDT-AuNPs, which identified dodecane as a decomposition product.[9] The peak near 722 cm^{-1} corresponding to the S-C stretch is not observed in the ODT-AuNP FTIR spectra, either due to low signal in this region or a chemical transformation of the ligand. Chemical transformation is supported by the peak near 1150 cm^{-1} (S=O stretching mode)[10], suggesting the oxidation of the sulfur, which was also observed in previous TGA-GC-MS studies of DDT-AuNPs.[9] Although the ligand does not contain oxygenated functional groups, evidence of remaining citrate after the ligand exchange is consistent with peaks near 1268 cm^{-1} (C-O stretching mode)[11], 1750 cm^{-1} (C=O stretching mode)[11], and 2300 cm^{-1} (C=O stretching mode of carbon dioxide).[10]

The FTIR spectra corresponding to the evolved gases at 470 °C are quite different than those of the evolved gases at 270 °C. Here, there is strong evidence for the decomposition of the ligand into 1-octadecene[10] based on peaks near 892 cm^{-1} & 970 cm^{-1} ($C{=}CH_2$ CH bending modes)[11], 1460 cm^{-1}(methylene scissoring mode)[11], 1644 cm^{-1} (C=C stretching mode)[11], 2800 cm^{-1} – 3000 cm^{-1} (methyl and methylene stretching modes)[11], and 3100 cm^{-1} ($C{=}CH_2$ CH stretching mode)[11]. The decomposition of bound octadecanethiol ligands to 1-octadecene is consistent with TGA-GC-MS studies of DDT-AuNPs, which identified 1-dodecene as a decomposition product[9]. As was seen in the FTIR spectra for the evolved gases at 270 °C, there is evidence of oxygenated functional groups in the spectra of the evolved gases at 470 °C that are likely due to species formed during the decomposition of remaining citrate. Here, peaks are seen near 1150 cm^{-1} (S=O stretching mode)[10], 1562 cm^{-1} (carboxylate stretching mode)[11], and 2300 cm^{-1} (C=O stretching mode of carbon dioxide)[10]. It is not surprising that the ligand exchange process did not remove all the original citrate ligands from the AuNP surfaces. Previous IR and XPS studies have suggested that hydrogen bonded networks of citrates on AuNP surfaces are resistant to desorption in the presence of alkanethiols[13]. Table S1 summaries the major peaks observed for the ODT-AuNP samples

Table S1. Assignments of major peaks observed in the FTIR spectra of evolved gases from the ODT-AuNP at 270 °C and 470 °C.

**Peak Position (cm^{-1})**	**Assignment**	**Likely Species**
	**270 °C**	
2800-3000	CH_3 and CH_2 stretching modes	Alkane and/or 1-octadecanethiol
2300	C=O stretching mode	Carbon dioxide from citrate decomposition
1750	C=O stretching mode	Product of citrate decomposition
1466	CH_2 scissoring mode	Alkane and/or 1-octadecanethiol
1386	CH_3 bending mode	Alkane and/or 1-octadecanethiol
1268	C-O stretching mode	Product of citrate decomposition
1150	S=O stretching mode	Product of citrate and ODT decomposition
	**470 °C**	
3100	$C=CH_2$ CH stretching mode	1-octadecene
2800 – 3000	CH_3 and CH_2 stretching modes	1-octadecene
2300	C=O stretching mode	Carbon dioxide from citrate decomposition
1644	C=C stretching mode	1-octadecene
1562	Carboxylate stretching mode	Product of citrate decomposition
1460	CH_2 scissoring mode	1-octadecene
1150	S=O stretching mode	Product of citrate and ODT decomposition
970 and 872	$C=CH_2$ CH bending modes	1-octadecene

The TGA-FTIR studies have provided strong evidence of different ODT ligand environments in the AuNP samples. The mass loss event near 270 °C likely corresponds to ODT ligands that are either free and/or loosely associated with the AuNP surface, while the mass loss event near 470 °C likely corresponds to ODT ligands that are tightly bound to the AuNP surface through a sulfur-gold bond. Our results are consistent with previous TGA-GC-MS studies of ~3 nm AuNPs functionalized with 1-DDT ligands[9]. These studies identified both strongly bonded ligands and additional multilayers weakly interacting with the particle surface. The studies presented here

demonstrate that 1-octadecanethiol was successfully added to the AuNP surfaces through the ligand exchange process. However, some original citrate ligands remained on the surface, likely through hydrogen bonding networks that were resistant to desorption. Importantly, the presence of remaining citrate ligands and free ODT ligands did not disrupt the biphasic monolayer adsorption assembly mechanism.

c. NMR Studies

The ODT-AuNPs were characterized using diffusion-ordered spectroscopy (DOSY), a 2-dimensional nuclear magnetic resonance (NMR) spectroscopy technique. All NMR solutions were prepared in $CDCl_3$. A Bruker Avance III spectrometer operating at a ^1H frequency of 400.18 MHz was used for all measurements. The operating temperature was 304 K. Standard 1-D ^1H measurements of ODT-AuNPs were 16 scans and a relaxation delay (D1) of 15 s. DOSY measurements were performed using Bruker pulse program *ledbpgp2s* with a relaxation delay (D1) of 15 s. The gradient strength was varied quadratically from 2 to 95% over 32 steps. The pulse length gradient (D20) and the gradient pulse duration (P30) were optimized to ensure the attenuation of the signal from the first to last spectrum was less than 10%. All DOSY 2-D spectral processing was performed using Mnova. All DOSY decay curves were plotted as ln (I/I_o) versus squared gradient field strength and fit in Igor using equation S1 or S2, a single or double exponential form of the Stejskal-Tanner equation, where D is the diffusion coefficient, g is the gradient field strength, γ is the gyromagnetic ratio, δ is the gradient pulse time, and Δ is the diffusion time.[14,15]

$$I = I_0 e^{-D\gamma^2 g^2 \delta^2 (\Delta - 0.6\delta)} \qquad \text{Eq. S1}$$

$$I = I_1 e^{-D_1\gamma^2 g^2 \delta^2 (\Delta - 0.6\delta)} + I_2 e^{-D_2\gamma^2 g^2 \delta^2 (\Delta - 0.6\delta)} \qquad \text{Eq. S2}$$

DOSY experiments were performed on 12, 19, 40, and 70 nm AuNPs functionalized with octadecanethiol. The alkyl, $\text{--}CH_2$, signal at 1.33 ppm (Figure S9) was chosen as the signal of interest for all DOSY decay curves.

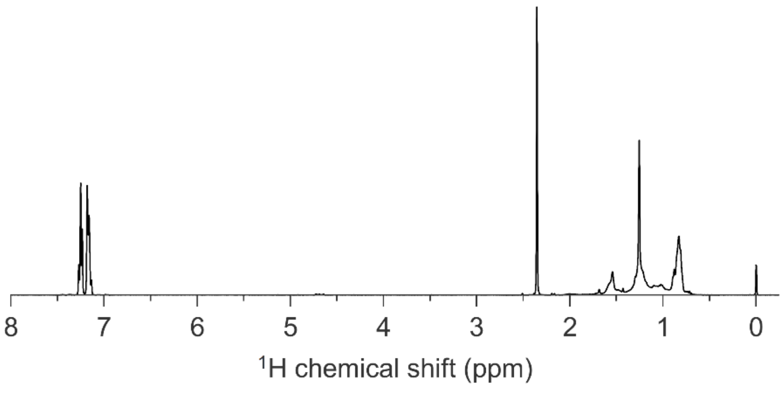

Figure S9. ^1H NMR spectrum of 19 nm ODT-AuNP in $CDCl_3$. The spectrum was collected using 16 scans and a D1 of 15 seconds. The signals at 0.83 ppm, 1.26 ppm, and 2.35 ppm correspond, respectively, to the $–CH_3$, $(\text{--}CH_2)_n$, and the $–CH_2$ (in the alpha position to the thiol). The $–CH_2$ in the beta position to the thiol appears at 1.58 ppm, but it is obscured by a small, but broad, peak at 1.54 ppm.

It was hypothesized that all four samples would exhibit DOSY data that would require a biexponential to be fit. Restated, it was hypothesized, based on the hyphenated TGA results, that both free and bound ligand would be observed. Figure S10 shows the resulting DOSY decay curves with their fit to either equation S1 or S2 (mono vs biexponential).[16]

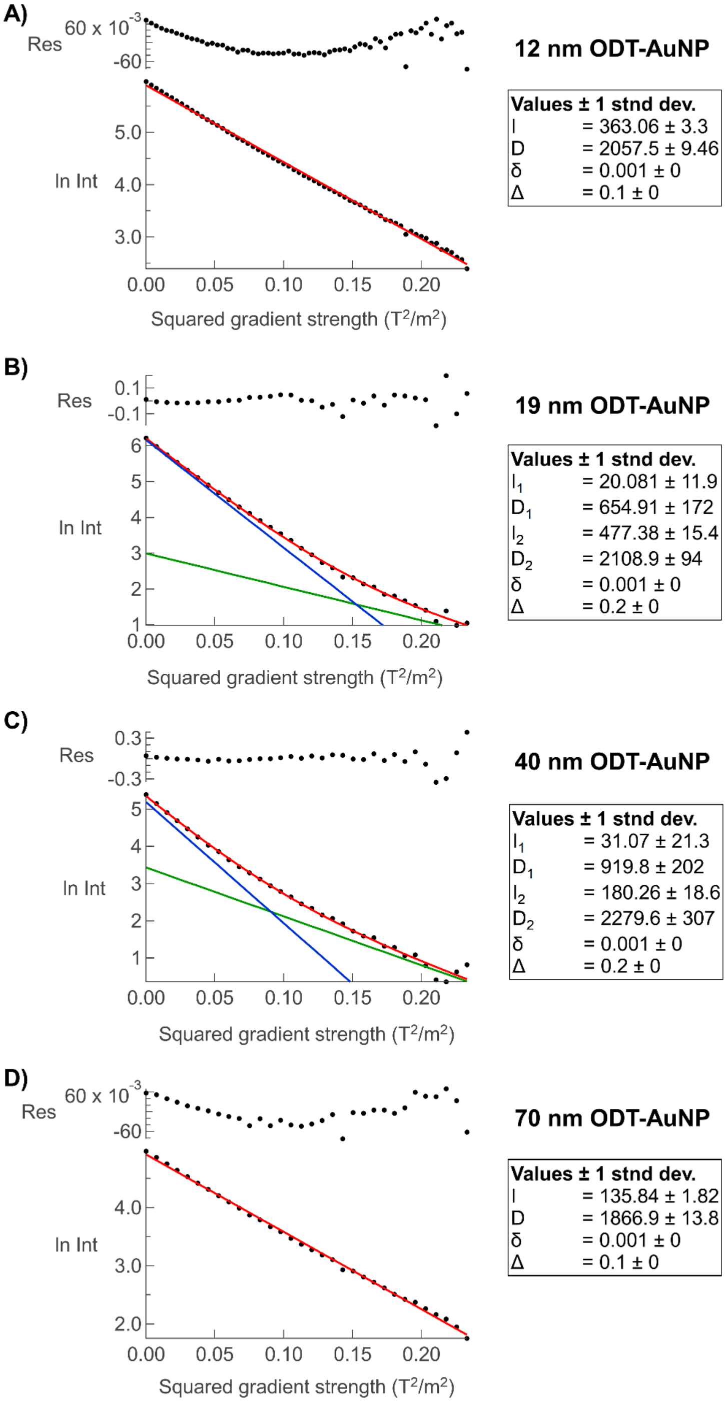

Figure S10. DOSY decay curves for (A) 12 nm, (B) 19 nm, (C) 40 nm, and (D) 70 nm ODT-AuNP. Decay curves were fit using a linearized form of either equation S1 or S2. The intensity at zero gradient field (*I*, unitless) and the diffusion coefficient (*D*, $\mu m^2/s$) were extracted from the fits. The gradient pulse time (δ, s) and the diffusion time (Δ, s) were input as given parameters from the data acquisition.

The ODT-AuNPs of size 19 nm and 40 nm exhibit the hypothesized DOSY decay data requiring a biexponential (eq S2), shown if Figure S9B and S9C, where two distinct diffusion coefficients are observed. These are indicative of free ODT and bound ODT. Meanwhile in Figure S10A and S10D, the 12 nm and 70 nm ODT-AuNPs, respectively, exhibit DOSY decay data fit with a single exponential (eq S1). The magnitude of the extracted diffusion coefficient (near 2000 $\mu m^2/s$) suggests that the signal is due to free ODT. In a comparison of the four DOSY decays, there is a diffusion coefficient indicative of free ODT, so it can be concluded that there is some amount of free ODT present in all of the samples. For the 12 nm and 70 nm ODT-AuNP samples, the signal from the free ODT dwarfs the potential signal from the bound ODT.

d. AuNP Visible Spectroscopy Data

Visible spectra (Figure S11) of the AuNPs before and after ligand exchange were collected using a VWR PV4 Visible Spectrophotometer at a resolution of 1 nm.

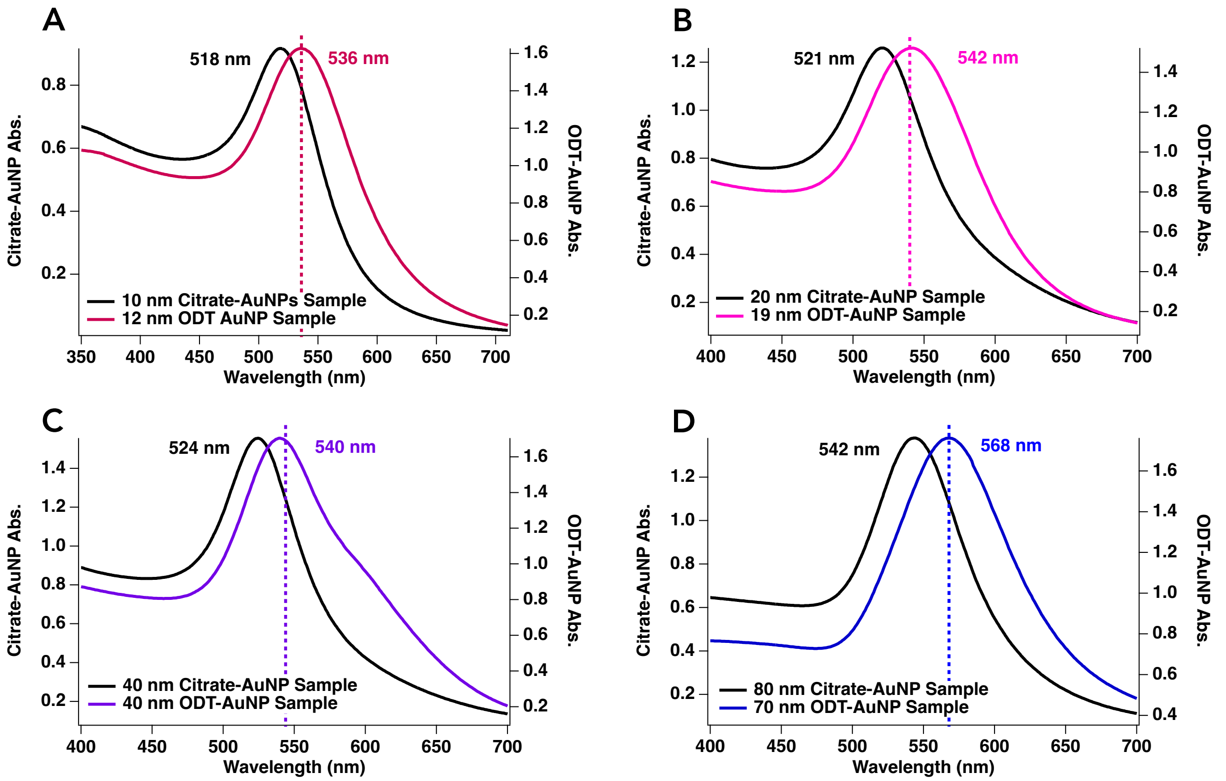

Figure S11. Visible spectra of AuNPs before (black traces) and after (color traces) functionalization with ODT ligands. (A) 10 nm citrate- and 12 nm ODT-AuNP samples; (B) 20 nm citrate- and 19 nm ODT-AuNP samples; (C) 40 nm citrate- and 40 nm ODT-AuNP samples; (D) 80 nm citrate- and 70 nm ODT-AuNP samples. The numbers on the graphs represent the experimental peak positions. The dashed lines represent expected peak positions from Mie Theory[17] after transferring the AuNPs from water to a toluene solution.

For all AuNP solutions, there was a redshift of the plasmonic peak upon AuNP functionalization with ODT and transfer from water to the organic phase. This redshift is expected due to the increase in refractive index of the solvent, which decreases the frequency of the AuNP localized surface plasmon resonance.[18] For water, the refractive index[19] is 1.33, for toluene[20] it is 1.50, and for chloroform[21] it is 1.44. Overall, the peak positions of the ODT-AuNP samples in toluene are consistent with those predicted by Mie Theory[17] (dashed lines on the graphs in Figure S11). The plasmonic peaks also visibly broaden after AuNP functionalization with ODT ligands. This broadening is indicative of an increase in the AuNP polydispersity and/or varying extents of agglomeration after hydrophobic functionalization.[18] These spectra are

consistent with the TEM images shown in Figure 2 of the main text, which show signs of agglomeration in the 12 nm and 19 nm ODT-AuNP samples, as well as multiple size distributions in the 40 nm and 70 nm ODT-AuNP samples.

The visible spectra of the ODT-AuNP samples were used to calculate estimated molar AuNP concentrations via Beer's Law based on the work on Haiss et. al.[22], who reported molar extinction coefficients of AuNPs at 450 nm for a range of diameters. Molar concentrations were converted to weight concentrations using AuNP volumes based on the average diameters and 19.28 g cm^{-1} as the density of gold.[22] Table S2 shows the average diameter for each ODT-AuNP sample, corresponding molar extinction coefficients, absorbances at 450 nm, calculated molar concentrations, and calculated weight concentrations. These calculated concentrations are rough estimates, as the ODT-AuNP samples likely contained agglomerates or multiple size distributions.

Table S2. Information used to calculate the estimated molar and weight concentrations.

Average ODT-AuNP Diameter (nm)	Molar Extinction Coefficient (M^{-1} cm^{-1})[22]	Absorbance at 450 nm	Calculated Molar Concentration (nM)	Calculated Weight Concentration (mg/mL)
12 ± 2	1.09×10^8	0.936	8.59	0.090
19 ± 2	4.60×10^8	0.803	1.75	0.073
40 ± 20	4.92×10^9	0.809	0.160	0.1
70 ± 30	2.71×10^{10}	0.743	0.0270	0.1

Experimental limitations did not make it feasible to achieve higher molar concentrations of ODT-AuNPs prepared from the 40 nm and 80 nm citrate-AuNPs. For these samples, transfer of the AuNPs from the aqueous phase to the chloroform phase after ODT functionalization was not as effective as it was for the 19 nm and 12 nm AuNP samples. Previous work has shown that as the AuNP diameter increases, alkanethiol ligands become less effective at sterically stabilizing AuNPs in hydrophobic solvents[23], thus increasing the propensity of the 40 nm and 80 nm ODT-AuNPs to adsorb to the chloroform-aqueous interface and stick to glassware. ODT-AuNPs at diameters greater than 80 nm could not be effectively functionalized and recovered. All experiments were thus carried out at a constant mass concentration rather than a constant molar concentration. Not only did this experimental challenge limit the concentrations of the larger AuNP samples but also did not allow for the identification a positive control that would disrupt nanosheet formation.

3. Supporting Electron Microscopy Data

a. Electron Beam Damage to the Nanosheets

The SEM images of the nanosheets made with the 19 nm, 40 nm, and 70 nm ODT-AuNP samples taken at high magnification (Figure S12) show that each AuNP and AuNP cluster are encased in a material that appears as a light gray halo. For the SEM images made with the 12 nm ODT-AuNP sample, halos were not observed at high magnification. At first, we attributed these halos in part to the peptoid monolayers separating from each other and encapsulating individual AuNPs and AuNP clusters.

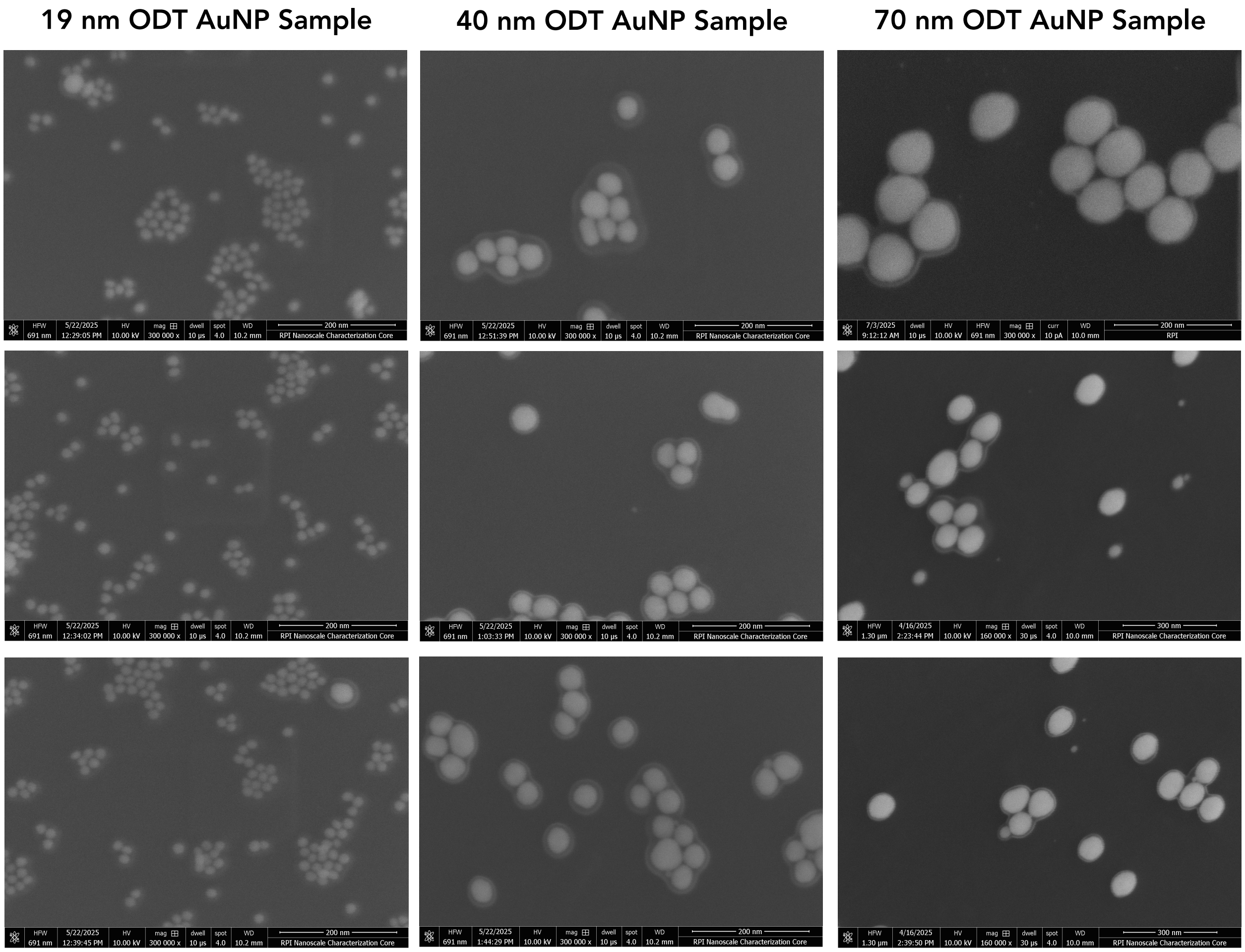

Figure S12. SEM images of AuNP nanosheets prepared with 19 nm (left column), 40 nm (middle column), and 70 nm (right column) ODT-AuNP samples. All images were taken at a magnification of 300,000 x.

To test our initial hypothesis, we performed a new experiment in which we prepared the peptoid nanosheets at the air-aqueous interface in which the peptoid was dissolved in a 0.05

mg/mL 40 nm citrate-AuNP solution. We hypothesized that forming the nanosheets in this way would lead to the AuNPs decorating the exteriors of the peptoid nanosheets. Figure S13 shows light microscope (A, B) and AFM (C, D) images of these nanosheets.

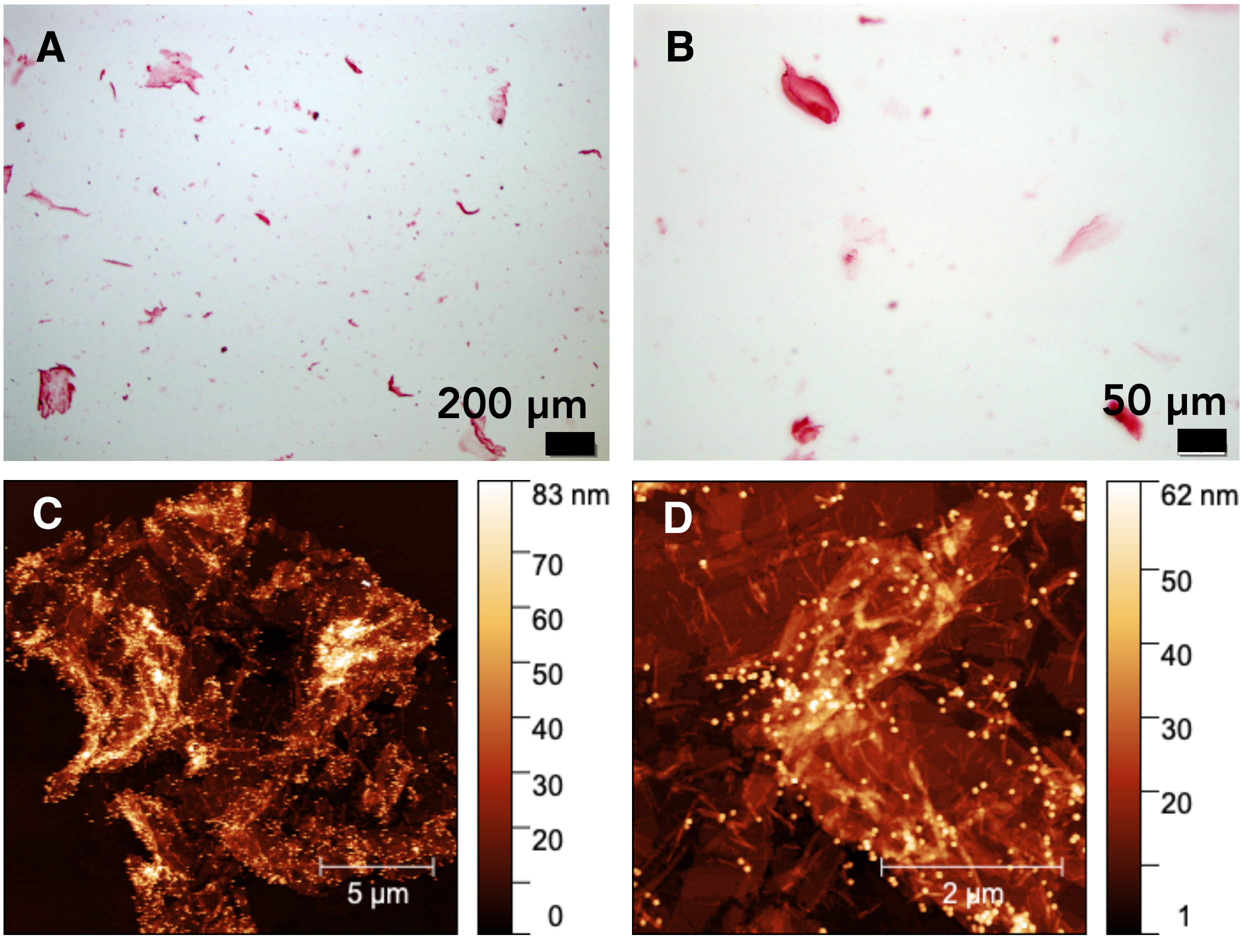

Figure S13. Light microscope (A, B) and AFM (C, D) images of peptoid nanosheets formed at the air-aqueous interface, in which the aqueous phase contained 0.05 mg/mL 40 nm citrate-AuNPs.

Upon collecting SEM images of these nanosheets (Figure S14), we observed the appearance of light gray halos around the AuNPs at 300,000 x magnification, as was observed in the ODT-AuNP nanosheets. These halos, however, were not initially present and only appeared within a second after focusing. This observation is consistent with damage to the organic material in the presence of the electron beam, as seen in previous electron microscopy studies of organic thin films.[24] We thus attribute the halos to the electron beam heating the AuNPs[25], which subsequently damages the material. Accelerating voltage as low as 5 kV was used to avoid beam damage but the halo still formed fast during image collection. Further lowering the voltage will deteriorate the resolution at this high magnification. To avoid beam damage, cryo-SEM needs to be used, which we do not have access to.

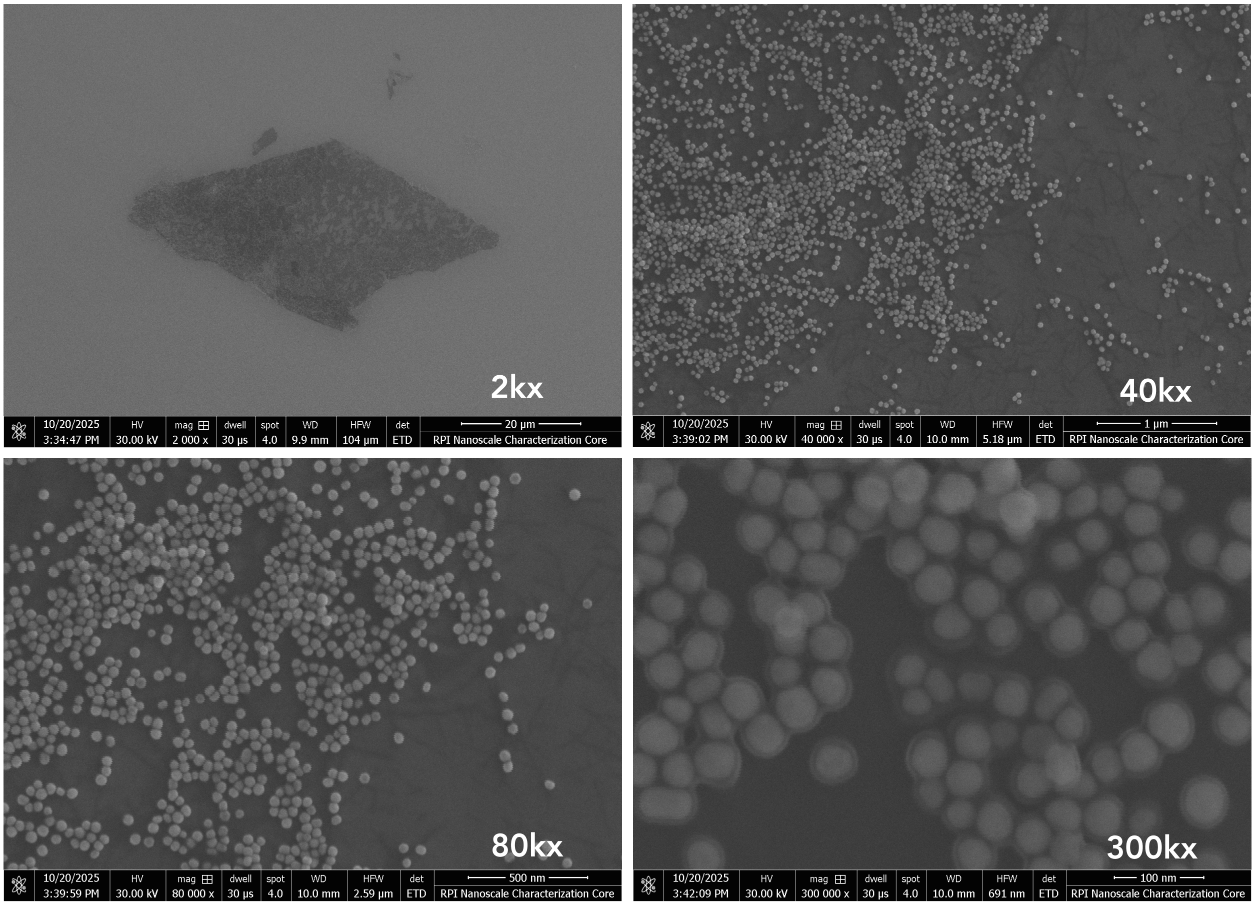

Figure S14. SEM images of peptoid formed at the air-aqueous interface, in which the aqueous phase contained 0.05 mg/mL 40 nm citrate-AuNPs.

b. TEM Images of the Gold Peptoid Nanosheets

Transmission electron microscope (TEM) studies of the gold peptoid nanosheets were performed by depositing 5 µL of the nanosheet solutions onto Carbon Type-B, 300 Mesh, Copper TEM grids from Ted Pella and collecting images using a JEOL 2011 TEM operated at 200 kV. Representative TEM images of the nanosheets are shown in Figure S15.

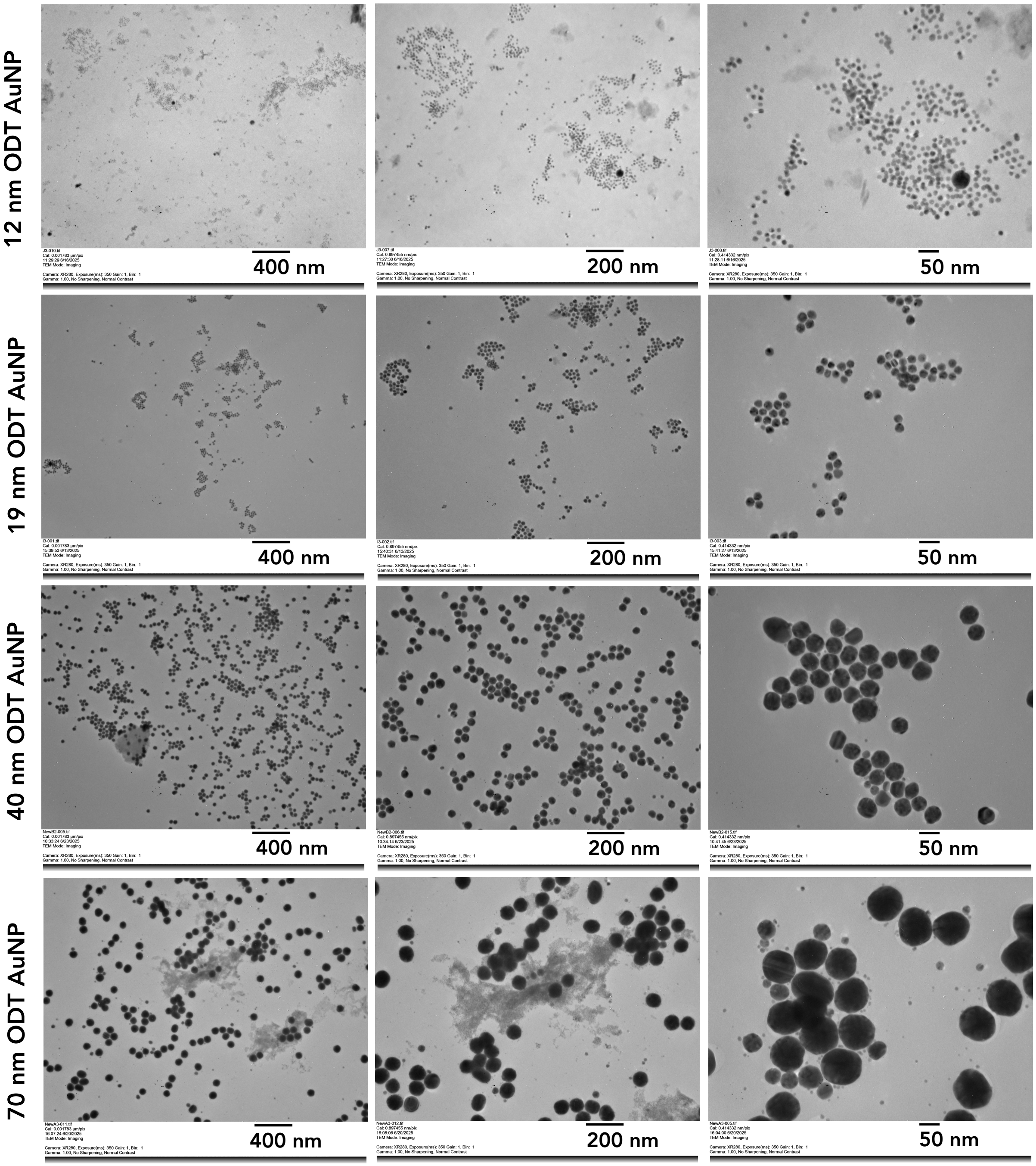

Figure S15. Representative TEM images of peptoid nanosheets prepared with 12 nm, 19 nm, 40 nm, and 70 nm ODT-AuNP samples.

There is not a clear contrast between the peptoid nanosheet and the TEM grid carbon support.

c. Agglomerated AuNPs

Several agglomerated particles were observed in SEM images of peptoid nanosheets prepared with 12 nm ODT-AuNPs. Examples of these agglomerates are shown in Figure S16.

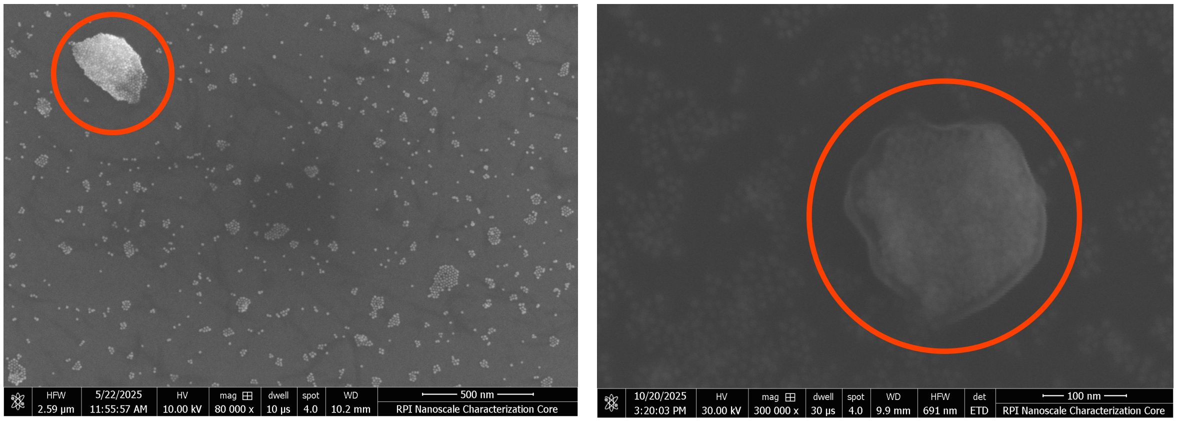

Figure S16. SEM image of agglomerated 12 nm ODT-AuNPs (circled) in the peptoid nanosheet.

The AuNP agglomerates are only a few hundreds of nanometers in diameter, which suggests that the upper size limit of cargo that can be incorporated into the nanosheets is beyond hundreds of nanometers.

4. References

(1) Soliwoda, K.; Tomaszewska, E.; Tkacz-Szczesna, B.; Mackiewicz, E.; Rosowski, M.; Bald, A.; Blanck, C.; Schmutz, M.; Novák, J.; Schreiber, F.; Celichowski, G.; Grobelny, J. Effect of the Alkyl Chain Length of Secondary Amines on the Phase Transfer of Gold Nanoparticles from Water to Toluene. *Langmuir* **2014**, *30* (23), 6684–6693. https://doi.org/10.1021/la501135q.

(2) Goulet, P. J. G.; Bourret, G. R.; Lennox, R. B. Facile Phase Transfer of Large, Water-Soluble Metal Nanoparticles to Nonpolar Solvents. *Langmuir* **2012**, *28* (5), 2909–2913. https://doi.org/10.1021/la2038894.

(3) McMahon, J. M.; Emory, S. R. Phase Transfer of Large Gold Nanoparticles to Organic Solvents with Increased Stability. *Langmuir* **2007**, *23* (3), 1414–1418. https://doi.org/10.1021/la0617560.

(4) Lista, M.; Liu, D. Z.; Mulvaney, P. Phase Transfer of Noble Metal Nanoparticles to Organic Solvents. *Langmuir* **2014**, *30* (8), 1932–1938. https://doi.org/10.1021/la404569h.

(5) Li, L.; Leopold, K.; Schuster, M. Comparative Study of Alkylthiols and Alkylamines for the Phase Transfer of Gold Nanoparticles from an Aqueous Phase to N-Hexane. *J. Colloid Interface Sci.* **2013**, *397*, 199–205. https://doi.org/10.1016/j.jcis.2013.01.062.

(6) Karg, M.; Schelero, N.; Oppel, C.; Gradzielski, M.; Hellweg, T.; von Klitzing, R. Versatile Phase Transfer of Gold Nanoparticles from Aqueous Media to Different Organic Media. *Chem. Eur. J.* **2011**, *17* (16), 4648–4654. https://doi.org/10.1002/chem.201003340.

(7) Cook, E.; Labiento, G.; Chauhan, B. P. S. Fundamental Methods for the Phase Transfer of Nanoparticles. *Molecules* **2021**, *26* (20), 6170. https://doi.org/10.3390/molecules26206170.

(8) Alkilany, A. M.; Caravana, A. C.; Hamaly, M. A.; Lerner, K. T.; Thompson, L. B. Phase Transfer of Citrate Stabilized Gold Nanoparticles Using Nonspecifically Adsorbed Polymers. *J. Colloid Interface Sci.* **2016**, *461*, 39–44. https://doi.org/10.1016/j.jcis.2015.09.010.

(9) Locardi, F.; Canepa, E.; Villa, S.; Nelli, I.; Lambruschini, C.; Ferretti, M.; Canepa, F. Thermogravimetry and Evolved Gas Analysis for the Investigation of Ligand-Exchange Reaction in Thiol-Functionalized Gold Nanoparticles. *J. Anal. Appl. Pyrolysis* **2018**, *132*, 11–18. https://doi.org/10.1016/j.jaap.2018.03.023.

(10) NIST Mass Spectrometry Data Center; Wallace, W. E. (director). Infrared Spectra. In *NIST Chemistry WebBook, NIST Standard Reference Database Number 69*; Linstrom, P. J., Mallard, W. G., Eds.; National Institute of Standards and Technology: Gaithersburg, MD, 20899.

(11) Crews, P.; Rodriguez, J.; Jaspars, M. *Organic Structure Analysis*; Oxford University Press: New York, NY, 1998.

(12) Marcilla, A.; Gómez-Siurana, A.; Beltrán, M.; Martínez-Castellanos, I.; Blasco, I.; Berenguer, D. TGA-FTIR Study of the Pyrolysis of Sodium Citrate and Its Effect on the Pyrolysis of Tobacco and Tobacco/SBA-15 Mixtures under N_2 and Air Atmospheres. *J. Sci. Food Agric.* **2018**, *98* (15), 5916–5931. https://doi.org/10.1002/jsfa.9121.

(13) Park, J.-W.; Shumaker-Parry, J. S. Strong Resistance of Citrate Anions on Metal Nanoparticles to Desorption under Thiol Functionalization. *ACS Nano* **2015**, *9* (2), 1665–1682. https://doi.org/10.1021/nn506379m.

(14) Stejskal, E. O.; Tanner, J. E. Spin Diffusion Measurements: Spin Echoes in the Presence of a Time-Dependent Field Gradient. *J. Chem. Phys.* **1965**, *42* (1), 288–292. https://doi.org/10.1063/1.1695690.

(15) Nilsson, M.; Connell, M. A.; Davis, A. L.; Morris, G. A. Biexponential Fitting of Diffusion-Ordered NMR Data: Practicalities and Limitations. *Anal. Chem.* **2006**, *78* (9), 3040–3045. https://doi.org/10.1021/ac060034a.

(16) De Roo, J. The Surface Chemistry of Colloidal Nanocrystals Capped by Organic Ligands. *Chem. Mater.* **2023**, *35* (10), 3781–3792. https://doi.org/10.1021/acs.chemmater.3c00638.

(17) Oldenburg, S. Light Scattering from Gold Nanoshells. Dissertation, Rice University, 2000.

(18) Ghosh, S. K.; Pal, T. Interparticle Coupling Effect on the Surface Plasmon Resonance of Gold Nanoparticles: From Theory to Applications. *Chem. Rev.* **2007**, *107* (11), 4797–4862. https://doi.org/10.1021/cr0680282.

(19) Thormählen, I.; Straub, J.; Grigull, U. Refractive Index of Water and Its Dependence on Wavelength, Temperature, and Density. *J. Phys. Chem. Ref. Data* **1985**, *14* (4), 933–945. https://doi.org/10.1063/1.555743.

(20) Debenham, M.; Dew, G. D. The Refractive Index of Toluene in the Visible Spectral Region. *J. Phys. E: Sci. Instrum.* **1981**, *14* (5), 544–545. https://doi.org/10.1088/0022-3735/14/5/004.

(21) Aminabhavi, T. M.; Banerjee, K. Density, Viscosity, Refractive Index, and Speed of Sound in Binary Mixtures of Dimethyl Carbonate with Methanol, Chloroform, Carbon Tetrachloride, Cyclohexane, and Dichloromethane in the Temperature Interfael (298.15-308.15) K. *J. Chem. Eng. Data* **1998**, *43* (6), 1096–1101. https://doi.org/10.1021/je980145.

(22) Haiss, W.; Thanh, N. T. K.; Aveyard, J.; Fernig, D. G. Determination of Size and Concentration of Gold Nanoparticles from UV−Vis Spectra. *Anal. Chem.* **2007**, *79* (11), 4215–4221. https://doi.org/10.1021/ac0702084.

(23) Zhang, S.; Leem, G.; Srisombat, L.; Lee, T. R. Rationally Designed Ligands That Inhibit the Aggregation of Large Gold Nanoparticles in Solution. *J. Am. Chem. Soc.* **2008**, *130* (1), 113–120. https://doi.org/10.1021/ja0724588.

(24) Leijten, Z. J. W. A.; Keizer, A. D. A.; De With, G.; Friedrich, H. Quantitative Analysis of Electron Beam Damage in Organic Thin Films. *J. Phys. Chem. C* **2017**, *121* (19), 10552–10561. https://doi.org/10.1021/acs.jpcc.7b01749.

(25) Fritsch, B.; Hutzler, A.; Wu, M.; Khadivianazar, S.; Vogl, L.; Jank, M. P. M.; März, M.; Spiecker, E. Accessing Local Electron-Beam Induced Temperature Changes during *in Situ* Liquid-Phase Transmission Electron Microscopy. *Nanoscale Adv.* **2021**, *3* (9), 2466–2474. https://doi.org/10.1039/D0NA01027H.
